# Catalytic Chan–Lam coupling using a ‘tube-in-tube’ reactor to deliver molecular oxygen as an oxidant

**DOI:** 10.3762/bjoc.12.156

**Published:** 2016-07-26

**Authors:** Carl J Mallia, Paul M Burton, Alexander M R Smith, Gary C Walter, Ian R Baxendale

**Affiliations:** 1 Department of Chemistry, Durham University, South Road, Durham, DH1 3LE, United Kingdom; 2 Syngenta, Jealott's Hill International Research Centre, Bracknell, Berkshire, RG42 6EY, United Kingdom

**Keywords:** Chan–Lam coupling, flow chemistry, gases in flow, oxygen, “tube-in-tube”

## Abstract

A flow system to perform Chan–Lam coupling reactions of various amines and arylboronic acids has been realised employing molecular oxygen as an oxidant for the re-oxidation of the copper catalyst enabling a catalytic process. A tube-in-tube gas reactor has been used to simplify the delivery of the oxygen accelerating the optimisation phase and allowing easy access to elevated pressures. A small exemplification library of heteroaromatic products has been prepared and the process has been shown to be robust over extended reaction times.

## Introduction

**Scheme 1:**
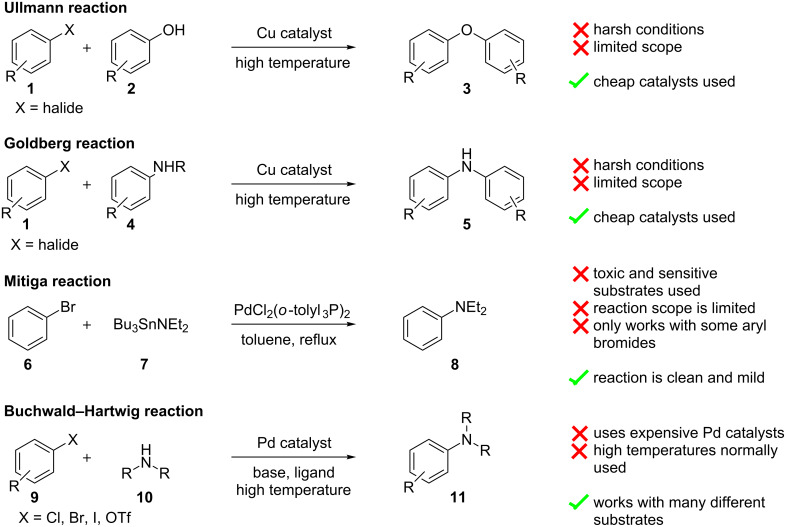
Comparison of early C–N and C–O coupling reactions.

The functionalisation of aromatic and aliphatic amines has received considerable attention due to the number of biologically active compounds represented by these classes. For this reason different synthetic methods for C–N bond formation have been developed (Scheme 1) over the years with the general goal to overcome the shortcomings of the original Ullman [1] and Goldberg [2] methods relating to the harsh reaction conditions they employ. After a closer look at the work of Mitiga [3] on the Stille coupling reactions, Hartwig [4] and Buchwald [5] independently proposed a catalytic mechanism and later reported a tin free aryl–amine coupling reaction [6–7]. This major breakthrough made the C–N coupling reaction accessible to a wide range of substrates, including anilines, which did not react very well with the previous conditions. However, despite the improvements achieved with the Buchwald–Hartwig coupling, limitations such as sensitivity to air and moisture, functional group tolerance and the high cost of palladium, reignited the search for an improved method.

In 1998, the groups of Chan [8], Evans [9] and Lam [10] independently reported upon mild methods for C(aryl)–N and C(aryl)–O coupling reactions. Their methods made use of stoichiometric amounts of copper(II) acetate as the catalyst and boronic acids as the aryl donors. In the presence of a base, the coupling could be performed at room temperature. These reactions were subsequently shown to work with a large number of nucleophiles and tolerated a variety of substrates, making the process one of the most efficient ways for C–N/O coupling [11]. Several modifications of the Chan–Lam reaction have been reported, expanding its scope and it has since been used to synthesise several biologically active compounds [11–12].

In 2009 the groups of Stevens and van der Eycken reported on the Chan–Lam reaction as a continuous flow protocol using copper(II) acetate (1.0 equiv), pyridine (2.0 equiv) and triethylamine (1.0 equiv) in dichloromethane [13]. Generally, when using anilines or phenols as the nucleophilic partner, moderate to good yields were obtained (56–71% yields, 9 examples). More recently the Tranmer group reported the use of a copper-filled column as a catalyst with TEMPO as the co-oxidant in acetonitrile (acetic acid additive) with moderate to good yields of the coupled products being obtained (25–79% yields, 16 examples) [14]. The use of a copper tubing which serves as both the reactor and the catalyst with *tert*-butyl peroxybenzoate as the oxidant in acetonitrile was also described but was outperformed by the copper filled column system. Although the use of elemental copper is potentially an improvement on the use of stiochiometric copper(II) acetate in continuous flow, the use of TEMPO or *tert*-butyl peroxybenzoate as a co-oxidant introduces waste. Employing oxygen gas as an oxidant is preferred as it is cheap, renewable and environmentally benign. We therefore set out to develop a more atom economical way of catalysing the Chan–Lam reaction using a sub-stoichiometric amount of copper and oxygen gas as the oxidant.

**Figure 1:**
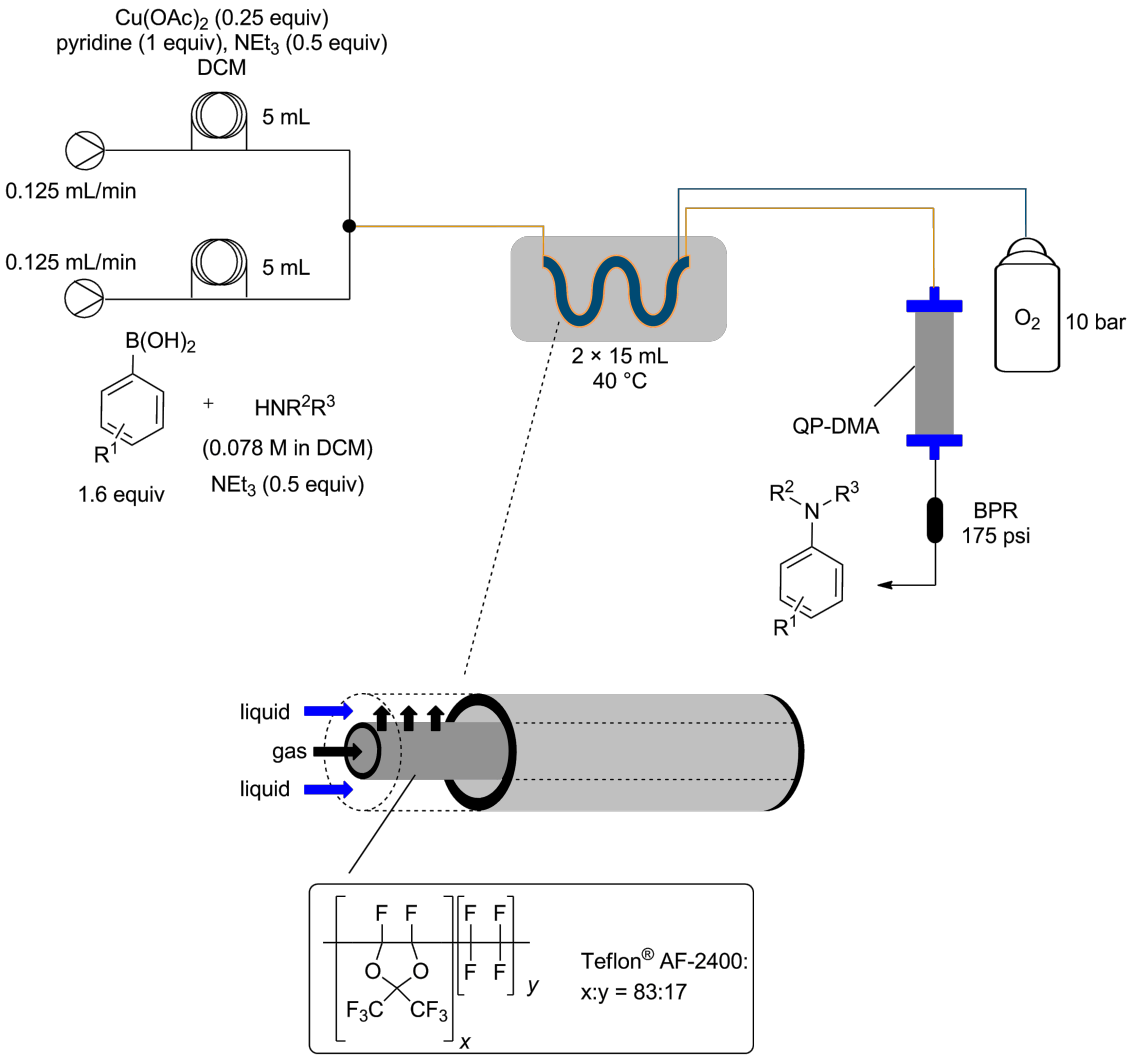
General flow scheme for catalytic Chan–Lam reaction.

The use of oxygen provides the necessary oxidant to reoxidise the Cu(I) that forms after the C–N reductive elimination back to Cu(II), allowing for sub-stoichiometric amounts of copper catalyst to be used [15–16]. Based upon our previous experience of using the reverse “tube-in-tube” reactor with other gases, it was decided that oxygen would be delivered via this reactor set-up (Figure 1).

## Results and Discussion

In our initial screening, four different organic solvents with good oxygen absorption were investigated (toluene, dichloromethane, acetonitrile and ethyl acetate), however, Cu(OAc)_2_ was only completely soluble in dichloromethane. Consequently dichloromethane was used as the reaction solvent. Unfortunately pumping dichloromethane through the HPLC pumps, used as part of the flow system, initially presented some issues. This was mainly due to cavitation which occurred just before the pump inlet, attributed to the shear forces present, causing outgassing (air). These bubbles, if allowed to enter the system disturbed the flow (or impaired the pump), resulting in unstable flow. The problem was solved when the dichloromoethane used was sonicated (30 min of sonication per 500 mL of solvent) prior to use, it was then maintained under positive pressure at the inlet throughout the experiment (N_2_ balloon was used for the positive pressure).

In an effort to identify the optimum conditions for the reaction process, the amount of copper catalyst and the oxygen pressure were studied (Table 1).

**Table 1:** Optimisation of the Chan–Lam reaction in continuous flow.

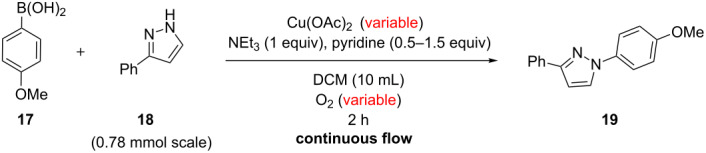					

Entry	Cu(OAc)_2_ (equiv)	Boronic acid (equiv)	Temperature (°C)	O_2_ pressure (bar)	NMR conversion (%)^a^

**1**	1.00	1.6	20	0	66

**2**	0.50	1.6	20	0	48

**3**	0.25	1.6	20	0	25

**4**	0.50	1.6	20	4	81

**5**	0.50	1.6	20	8	85

**6**	0.50	1.6	20	10	97

**7**	0.50	1.6	20	12	85

**8**	0.50	1.6	20	14	83

**9**	0.25	1.6	20	10	94

**10**	0.25	1.6	20	12	87

**11**	0.10	1.6	20	10	50

**12**	0.25	1.4	20	10	56

**13**	0.25	1.1	20	10	48

**14**	0.25	1.6	30	10	87

**15**	0.25	1.6	40	10	95

**16**	0.25	1.6	50	10	88

**17**^b^	0.25	1.6	40	10	93

**18**^c^	0.25	1.6	40	10	76

^a^Yields calculated using 1,3,5-trimethoxybenzene as an internal NMR standard and represents the average of two runs. ^b^1.5 equiv of pyridine, ^c^0.5 equiv of pyridine.

**Figure 2:**
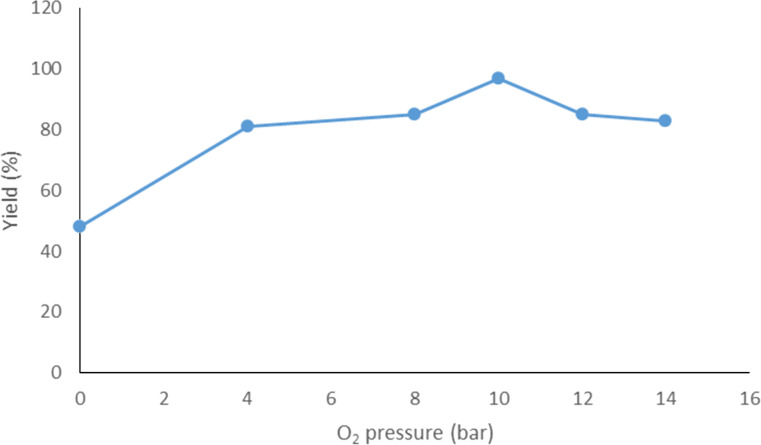
Observed trend for the effect of changing oxygen pressure on the NMR yield of **19**.

A set of control experiments with no oxygen was run and the amount of copper acetate catalyst was lowered from 1 to 0.25 equiv (entries 1–3, Table 1). As anticipated, with no oxidant to reoxidize the catalyst, the yield of **19** dropped in proportion to the amount of catalyst used. Next, whilst maintaining the amount of copper acetate (0.5 equiv), the effect of the oxygen pressure on conversion was investigated (entries 4–8, Table 1). A general increase in the yield of **19** was obtained on going from atmospheric to 10 bar after which a slight decrease in yield was encountered at higher pressures (Figure 2). This same decrease in yield was also observed when going from 10 bar to 12 bar of oxygen using 0.25 equiv of copper acetate (entries 9 and 10, Table 1).

When the amount of copper acetate was reduced to 0.1 equiv a drastic decrease in yield was observed indicating that the TOF of the catalyst prevented achievement of good yields within the time limits (residence time) of the flow reactor (entry 11, Table 1). A decrease in yield was also observed when the amount of boronic acid used was decreased to 1.4 equiv and 1.1 equiv, respectively (entries 12 and 13, Table 1). Changing the temperature from 20 °C to 50 °C did not greatly affect the yields obtained, with 40 °C giving the most promising result (entries 14–16, Table 1). However, it was observed that less particulate matter was formed in the reactor when higher temperatures were used (40 and 50 °C), which helps in avoiding possible reactor blockages. Finally, the amount of pyridine added was also studied. Decreasing the amount of pyridine (0.5 equiv, entry 18, Table 1) resulted in a lower yield (76%) while increasing the amount of pyridine (1.5 equiv, entry 18, Table 1) did not produce any noticeable change in the yield (93%). This indicates that the pyridine plays an important role in this coupling reaction which could be both due to its effect as a ligand and/or its solubility enhancement of the copper acetate. The amount of triethylamine was not varied as its quantity was required to ensure the boronic acid remained soluble in the dichloromethane solvent.

To determine the time needed to reach steady state in the reactor, samples were periodically collected (every 2 min via an autosampler) and analysed by ^1^H NMR spectroscopy using 1,3,5-trimethoxybenzene as an internal standard. As expected, the product started eluting after 120 min which corresponds with the theoretical residence time. A lower yield was initially obtained for 120 min (85% yield) which then rapidly increased to 98% yield at 125 min. The yield then stabilised from 135 min at 96% indicating steady state was achieved.

**Figure 3:**
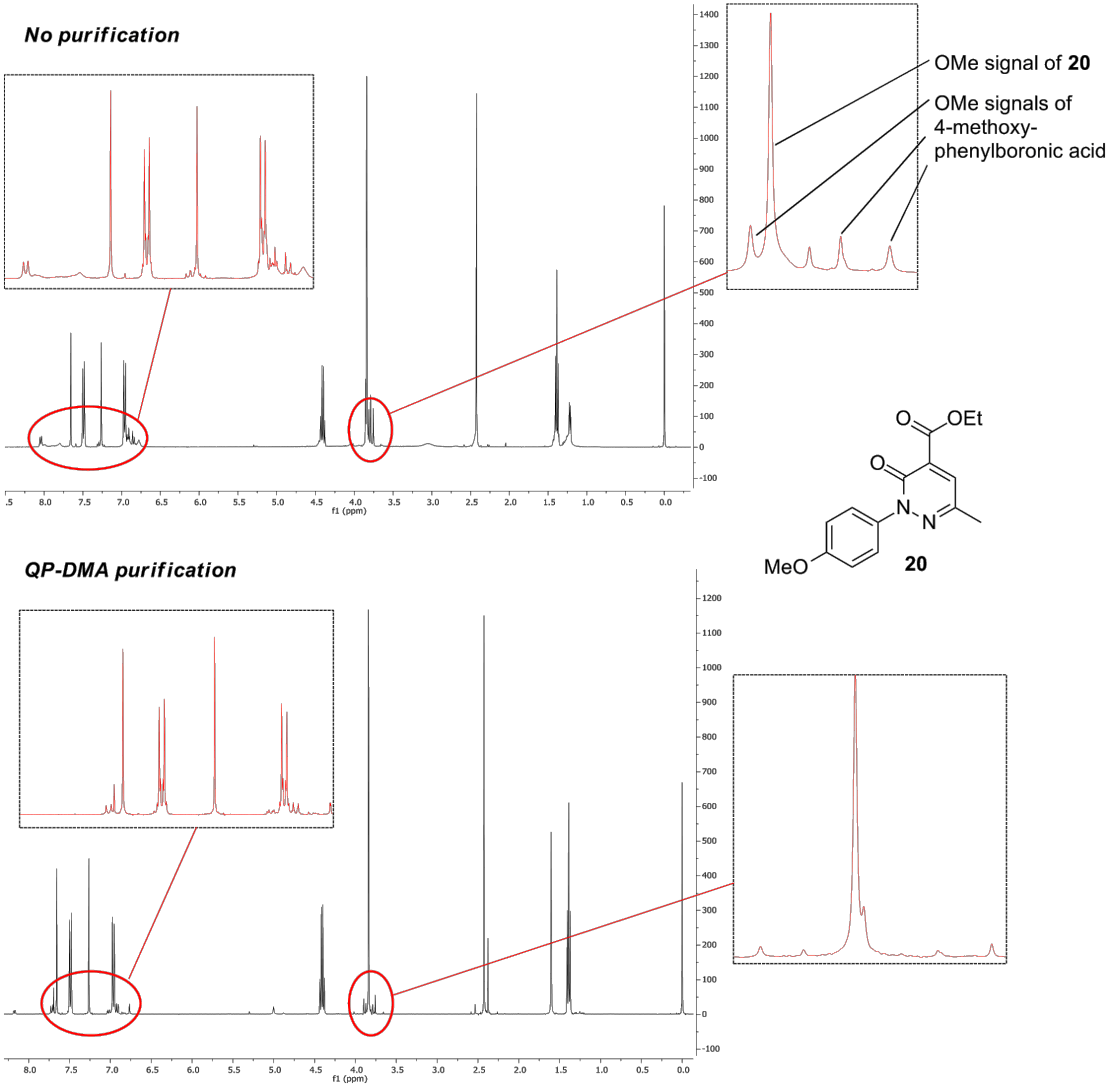
Comparison of ^1^H NMR spectra of non-purified (top) and QP-DMA purified (bottom) continuous flow synthesis of compound **20**.

As it had been determined that the amount of arylboronic acid excess could not be lowered (entries 12 and 13, Table 1), the use of a polymer supported scavenger was tested in an effort to sequester the excess boronic acid. A column of QP-DMA, a polymer-supported tertiary amine base, was placed in-line after the “tube-in-tube” reactor (Figure 1). It was found that this was sufficient to remove the majority of boronic acid without affecting the yield of the product (Figure 3). Ultimately as the products were required for biological screening they were still purified by column chromatography, however, the reduction of the boronic acid excess made the chromatography far easier.

### Reaction scoping and library preparation

**Scheme 2:**
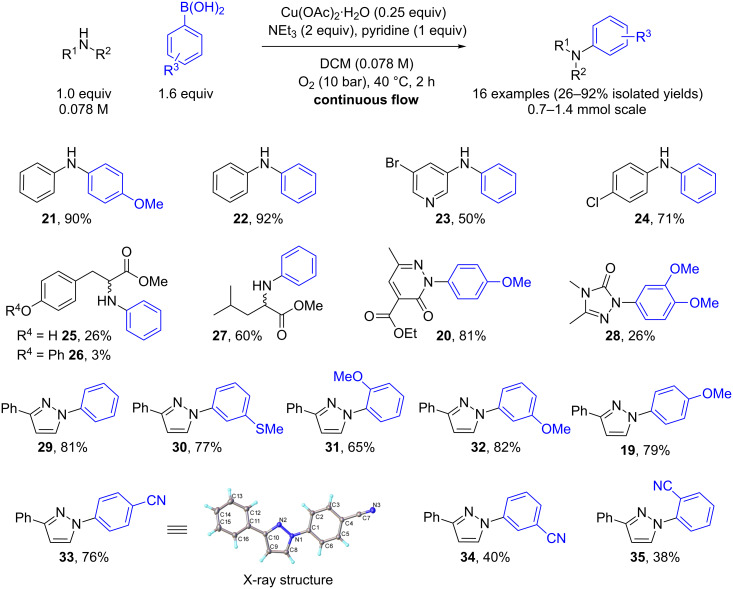
Scope of the catalytic Chan–Lam reaction in continuous flow.

Using the optimised conditions determined for the synthesis of compound **19**, a small library was prepared to demonstrate the scope of the reaction conditions. Excellent isolated yields were obtained when anilines were used as the nucleophilic partner with both 4-methoxyphenylboronic acid (90% yield of **21**) and phenylboronic acid (92% yield of **22**) as the aryl donors (Scheme 2). Phenylboronic acid also gave a moderate isolated yield when coupled with 3-amino-5-bromopyridine as the nucleophile (50% yield of **23**, Scheme 2) and a good isolated yield with the electron withdrawing 4-chloroaniline (71% yield of **24**, Scheme 2). Using *L*-tyrosine methyl ester as the nucleophile with phenylboronic acid, unfortunately, gave a poor isolated yield of 26% and also underwent some epimerisation (**25**, 53% ee determined by chiral HPLC, Scheme 2). Additionally, a small amount of the product (**25**) reacted further with phenylboronic acid through the phenol to give **26** in 3% isolated yield. In the case of *L*-leucine methyl ester an isolated yield of 60% was realised, but this substrate also underwent partial epimerisation (**27**, 71% ee determined by chiral HPLC, Scheme 2).

**Scheme 3:**
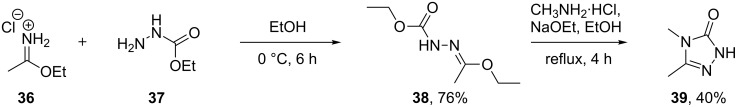
Syntheses of substrate **39**.

Using *N*-heterocyclic substrates as the nucleophilic partner with a range of different phenylboronic acids generally gave good isolated yields (**19**, **20**, **28–35**, Scheme 2). Using a pyradizine as a nucleophilic partner an 81% yield was obtained for the formation of **20**. However, using 3,4-dimethyl-1*H*-1,2,4-triazol-5(4*H*)-one (**39**), which was synthesised using a literature procedure [17–18] (Scheme 3), with 3,4-dimethoxyphenylboronic acid gave a lower yield of 26% (**28**, Scheme 2). It is not yet clear as to why such a low conversion and isolated yield was obtained although the reduced nucleophilicity and higher potential for coordination of the triazole to the copper catalyst might inhibit catalyst turnover and account for this.

Alternatively, using 3-phenyl-1*H*-pyrazole (**18**) as the nucleophile with a number of different phenylboronic acids gave moderate to good yields (38–82% yields). In general electron-rich phenylboronic acids (**19**, **29–32**, Scheme 2) gave better yields than electron poor ones (**33**–**35**, Scheme 2). This is probably due to the more favourable thermodynamics with an increase in the electropositive nature of boron, which in turn increases the rate of the transmetallation step. Changing the group at the 4-position of the phenylboronic acid gave good yields for both electron-rich (**19**, 79% yield) and electron-poor (**33**, 76% yield) phenylboronic acids. On the other hand changing the group at the 3-position of the phenylboronic acid gave good yields for electron-rich (**30** and **32**, 77% and 82% yields, respectively) but only a moderate yield of 40% for electron-poor (**34**) phenylboronic acids. Lower yields were also encountered for both electron-rich (65% yield) and electron-poor (38% yield) 2-substituted phenylboronic acids, most likely due to steric factors (**31** and **35**, Scheme 2).

**Figure 4:**
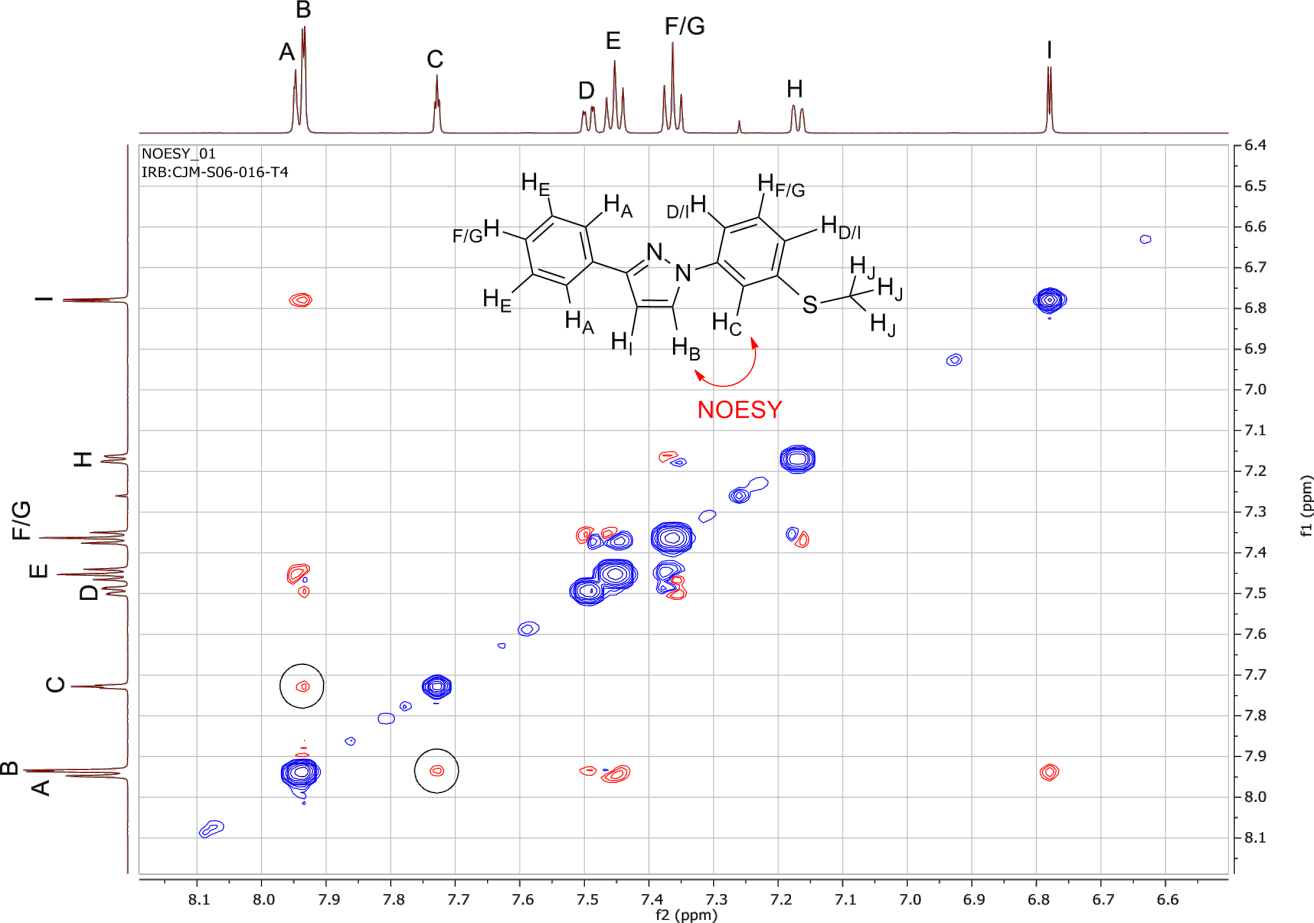
NOESY NMR spectrum for **30** with the characteristic NOESY signal encircled.

**Figure 5:**
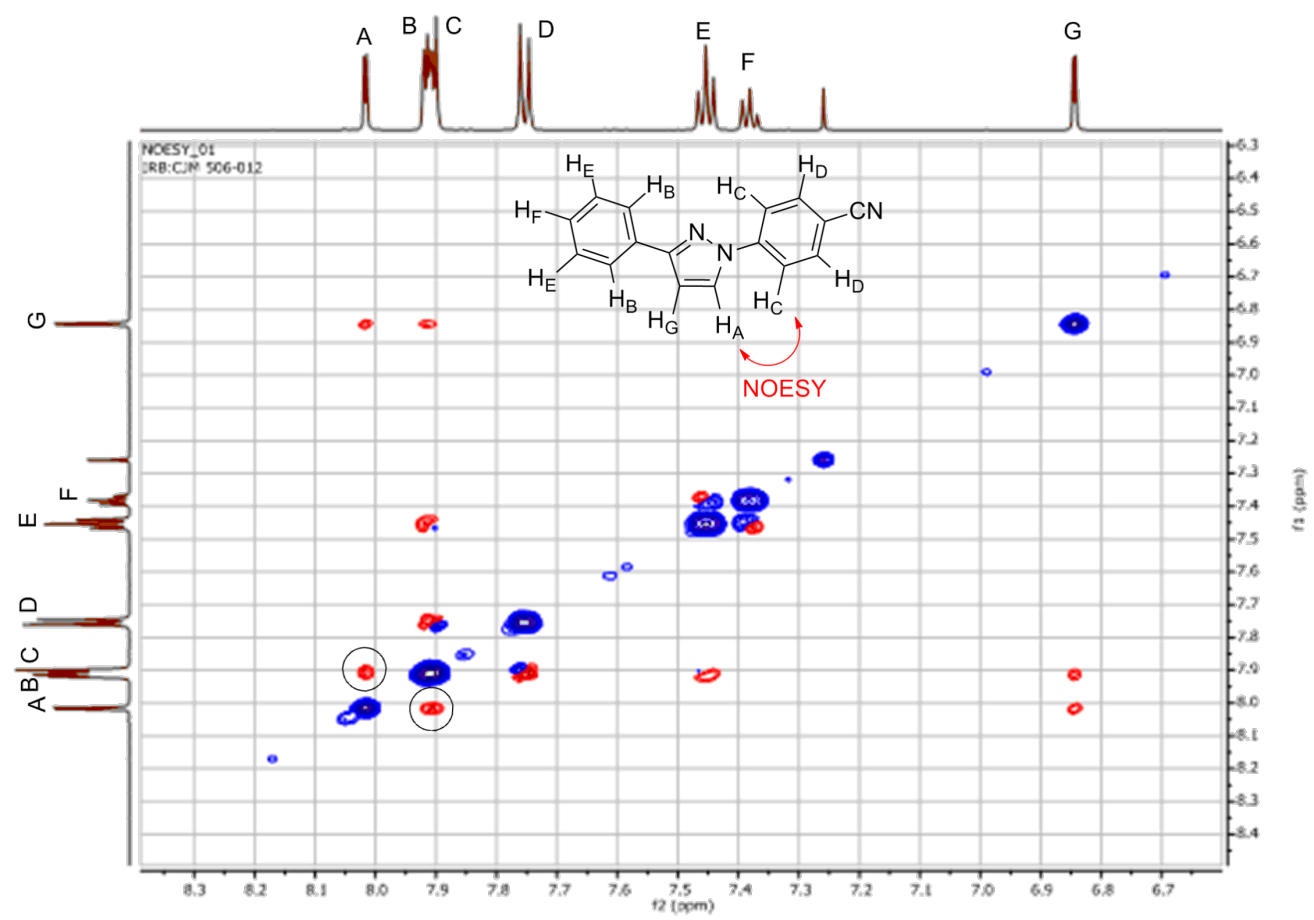
NOESY NMR spectrum for **33** with the characteristic NOESY signal encircled.

**Figure 6:**
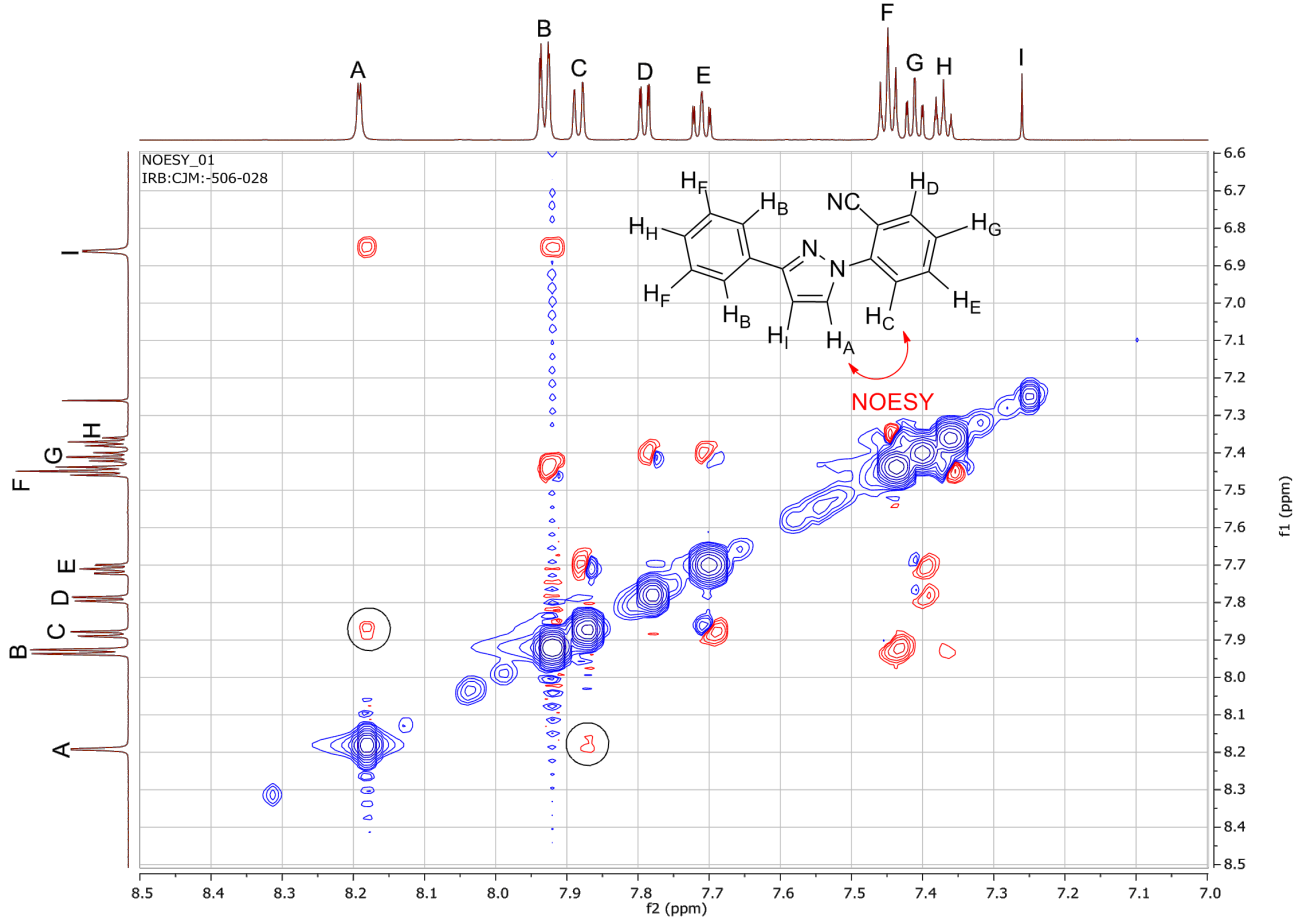
NOESY NMR spectrum for **35** with the characteristic NOESY signal encircled.

It is noteworthy that for all of the 3-phenyl-1*H*-pyrazole couplings, only the 1,3-disubsituted pyrazole products were obtained with no 1,5-disubsituted isomers being detected. The regioselectivity of the 1,3-disubsituted pyrazoles was confirmed by NOESY NMR experiments (**30**, **33** and **35**, Figures 4–6) as well as comparison to known published data. In addition, an X-ray crystal structure for compound **33** was obtained and the connectivity confirmed. It was noted that several examples of literature reported cases where mixtures of regioisomers had been obtained were wrongly assigned.

**Figure 7:**
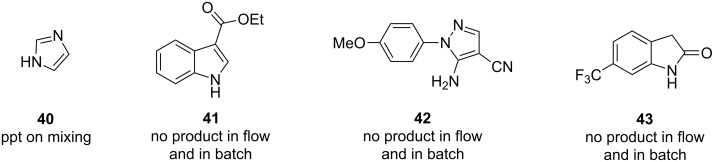
Substrates that gave no products in flow.

The process described does have certain limitations. For certain nucleophilic substrates no products were obtained when C–N coupling with 4-methoxyphenylboronic acid (**17**) was attempted (Figure 7). In the case of substrate **40** precipitation occurred as soon as the two solutions came into contact at the T-piece mixer, which was probably due to strong coordination to the copper acetate by the imidazole ring. This made running this reaction problematic in flow due to the occurrence of reactor blocking. Other substrates proved unreactive. In the case of starting materials **41–43** the reduced nucleophilicity of these substrates might account for the lack of conversion. By comparison, all three substrates (**41–43**) also failed to react under batch conditions using 2 equiv of Cu(OAc)_2_, 2 equiv of NEt_3_ and 1 equiv of pyridine at 40 °C for 48 h confirming their low reactivity.

### Reaction scaling

**Scheme 4:**
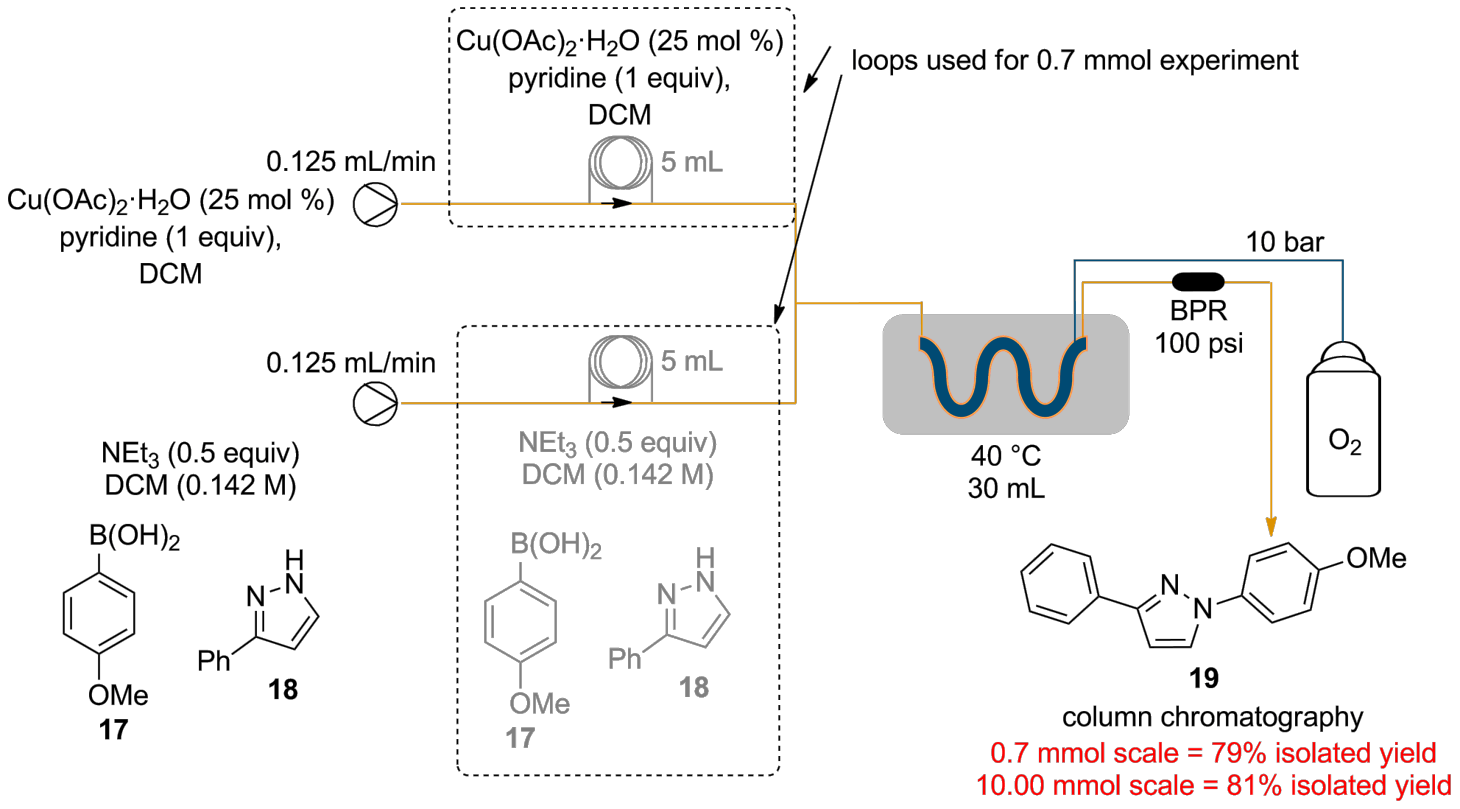
Scale-up procedure for **19**.

Finally, the robustness of the process and potential for scalability of the general reaction conditions was demonstrated by the synthesis of **19** at a 10 mmol scale, a factor of fourteen times the original 0.7 mmol test reaction (Scheme 4). A slightly improved isolated yield (81%) was obtained for the larger scale experiment when compared to the 79% isolated yield obtained for the shorter run experiment. The consistency of the yields obtained indicates that the process is robust and without modification can reliably deliver 0.216 g h^−1^ of **19** at 81% isolated yield.

## Conclusion

The use of flow chemistry for the C–N coupling through a catalytic Chan–Lam reaction has allowed for a safe and efficient introduction of oxygen through a reverse “tube-in-tube” reactor. Optimisation of the reaction conditions allowed for a scalable and efficient way for the continuous synthesis of a number of functionalised aromatic and aliphatic amines including a number of 1,3-disubstituted pyrazoles which were selectively obtained over the regioisomeric 1,5-disubstituted products. When compared to other published protocols it is clear that the use of sub-stoichiometric amounts of the copper catalysts presents an advantage over the stoichiometric amount used in the original flow studies [13]. Additionally, the use of oxygen as the oxidant offers improved atom economy over the use of systems such as TEMPO and *tert*-butyl peroxybenzoate [14]. We believe this approach therefore present several opportunities for laboratory chemists to utilise this valuable C/N coupling methodology.

## Experimental

### Warning: Oxygen is a highly flammable gas and all reactions were carried out in well ventilated fume cupboards

For the flow process, 0.781 mmol of the amine was dissolved in 5.5 mL of dichloromethane followed by 1.25 mmol of the boronic acid and NEt_3_ (0.039 g, 54 µL, 0.391 mmol). Another solution containing Cu(OAc)_2_·H_2_O (0.195 mmol, 0.25 equiv), NEt_3_ (0.039 g, 54 µL, 0.391 mmol) and pyridine (0.062 g, 63 µL, 0.781 mmol) in 5.5 mL of dichloromethane was also prepared. The two solutions were separately introduced in a 5 mL loop as shown in Table 1. The pumps were each set at 0.125 mL/min to achieve a residence time of 2 h. Two reverse “tube-in-tube” reactors (supplied by Vapourtec) were used in series to achieve a combined reactor volume of 30 mL which were heated at 40 ºC. The reaction mixture was then passed through an Omnifit column (r = 0.33 cm, h = 10.00 cm) filled with QP-DMA followed by a back pressure regulator (175 psi). The crude reaction mixture was then passed through a plug of silica to remove most of the excess copper present and the organic solvent from eluent evaporated under reduced pressure. The resultant crude material was then purified using flash chromatography.

## Supporting Information

File 1 Experimental procedures and characterization data for all new compounds.
