# Supplementary material for: Catalytic Chan–Lam coupling using a ‘tube-in-tube’ reactor to deliver molecular oxygen as an oxidant
Source: Beilstein J Org Chem. 2016 Jul 26;12:1598–607. doi: 10.3762/bjoc.12.156 (PMC4979635; doi:10.3762/bjoc.12.156)
Supplement: File 1 — Experimental procedures and characterization data for all new compounds. [file Beilstein_J_Org_Chem-12-1598-s001.pdf]

Supporting Information

for

Catalytic Chan–Lam coupling using a 'tube-in-tube' reactor to deliver molecular oxygen as an oxidant

Carl J. Mallia[1], Paul M. Burton[2], Alexander M. R. Smith[2], Gary C. Walter[2] and Ian R. Baxendale*[1]

Address: [1]Department of Chemistry, Durham University, South Road, Durham, DH1 3LE, United Kingdom and [2]Syngenta, Jealott's Hill International Research Centre, Bracknell, Berkshire, RG42 6EY, United Kingdom

Email: Ian R. Baxendale - i.r.baxendale@durham.ac.uk

*Corresponding author

Experimental procedures and characterization data for all new compounds

Unless specified, reagents were obtained from commercial sources and used without further purification. Solvents were obtained from Fisher scientific, and H_2O was deionised before use.

NMR spectra were recorded on either Bruker Avance-400, Varian VNMRS-600 or Varian VNMRS-700 instrument and was calibrated to the residual solvent according to the literature [1]. Assignments are based on DEPT-135, COSY, NOESY, HSQC and HMBC spectra.

Liquid chromatography-mass spectrometry (LCMS) was performed on an Agilent HP 1100 series chromatograph (Mercury Luna 3µ C18 (2) column) attached to a Waters ZQ2000 mass spectrometer with ESCi ionisation source in ESI mode. Elution was carried out at a flow rate of 0.6 mL/min using a reverse phase gradient of MeCN–water containing 0.1% formic acid. Gradient = 0–1 min: hold MeCN 5%, 1–4 min: ramp MeCN 5–95%, 4–5 min: hold MeCN 95%, 5–7 min: ramp MeCN 95–5%, 7–8 min: hold MeCN 5%. Retention times are reported as Rt.

High resolution mass spectra (HRMS) were recorded on a Waters Micromass LCT Premier spectrometer using time of flight with positive electrospray ionisation (ESI+), an ABI/MDS Sciex Q-STAR Pulsar with ESI+ and an ASAP (atmospheric pressure solids analysis probe ionisation), or a Bruker BioApex II 4.7e FTICR utilising either ESI+ or a positive electron ionisation (EI+) source equipped with a direct insertion probe. The mass reported is that containing the most abundant isotopes (^{35}Cl and ^{79}Br). Limit: ± 5 ppm.

IR spectra were recorded neat on a Perkin-Elmer Spectrum Two FTIR spectrometer using Universal ATR sampling accessories. Letters in parentheses refer to the relative absorbency of the peak: w – weak (<40% of the most intense peak), m – medium (40–75% of the most intense peak), s – strong (>75% of the most intense peak) and br – broad.

Melting points were recorded on an Optimelt automated melting point system with a heating rate of 1 °C/min (70% onset point and 10° clear point) and are uncorrected.

X-ray diffraction experiment for **33** was carried out on a D8 Venture 3-circle diffractometer (Bruker AXS) with a PHOTON 100 CMOS area detector, using Mo Kα radiation ($\bar{\lambda}$ =0.71073 Å) from an IµS microsource with focusing mirrors. The crystal was maintained at T=120 K using a Cryostream (Oxford Cryosystems) open-flow N_2 cryostat. The structure was solved by direct methods and refined by full-matrix least squares against F^2 of all reflections, using OLEX2 [2], SHELXS 2013/1 [3] and SHELXL 2014/7 [4] software. Crystallographic data for structure **33** have been deposited with the Cambridge Crystallographic Data Centre as supplementary publication CCDC-1479349.

A) Catalytic Chan–Lam in flow:

A solution was prepared from the amine (0.781 mmol) in DCM (5.5 mL) and the boronic acid (1.25 mmol) and NEt_3 (0.039 g, 54 µL, 0.391 mmol) were added. A second solution was prepared with $Cu(OAc)_2 \cdot H_2O$ (0.195 mmol, 0.25 equiv), NEt_3 (0.039 g, 54 µL, 0.391 mmol) and pyridine (0.062 g, 63 µL, 0.781 mmol) in DCM (5.5 mL). The two solutions were introduced to independent 5 mL sample loop as shown in (Scheme 1). The dispensing HPLC pumps were each set at 0.125 mL/min to achieve a residence time of 2 h. Two reverse “tube-in-tube” reactors were used in series to achieve a combined reactor volume of 30 mL which were heated at 40 °C. The reaction mixture was then passed through an Omnifit column (r = 0.33 cm, h = 10.00 cm) filled with QP-DMA followed by a back pressure regulator (175 psi). The crude reaction mixture was passed through a plug of silica to remove base line residue and the solvent evaporated under reduced pressure. The resultant crude material was then purified using flash chromatography.

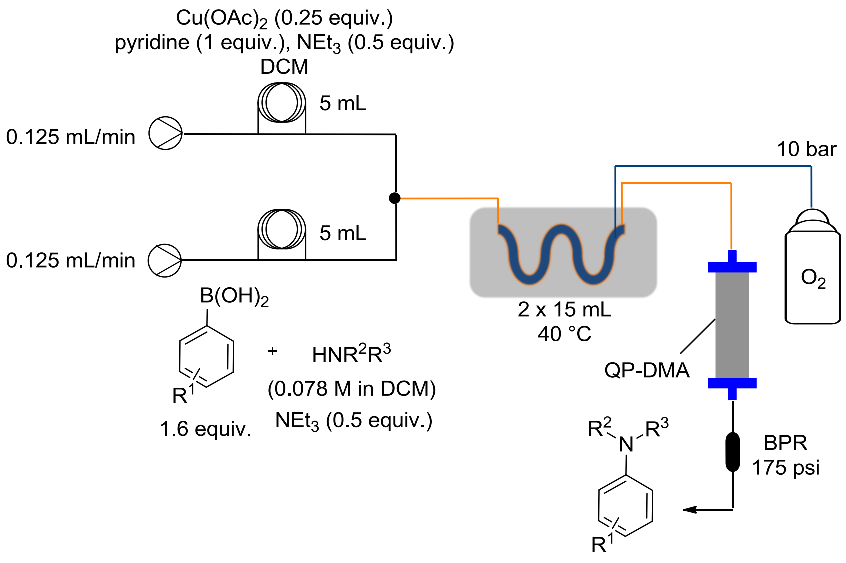

Scheme 1: General flow scheme for catalytical Chan Lam reaction.

Spectroscopic and experimental data:

1-(4-Methoxyphenyl)-3-phenyl-1*H*-pyrazole, 19:

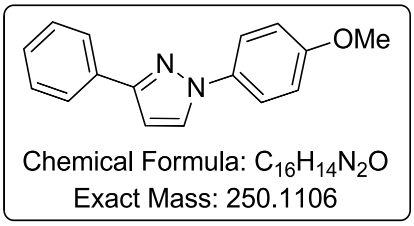

Consistent with published data [5].

Prepared using general procedure A: Isolated yield; 0.139 g (79%, 0.70 mmol scale);

Colourless crystals; Rf: 0.36 (8/2, EtOAc/hexanes);

^{1}H NMR (700 MHz, $CDCl_3$) 7.94 – 7.90 (m, 2H), 7.85 (d, J = 2.5 Hz, 1H), 7.69 – 7.65 (m, 2H), 7.46 – 7.41 (m, 2H), 7.34 (ddt, J = 7.4, 5.6, 1.3 Hz, 1H), 7.01 – 6.97 (m, 2H), 6.75 (d, J = 2.4 Hz, 1H), 3.85 (s, 3H);

^{13}C NMR (176 MHz, $CDCl_3$) δ/ppm 158.3 (C), 152.7 (C), 134.2 (C), 133.4 (C), 128.8 (CH), 128.2 (CH), 128.0 (CH), 125.9 (CH), 120.9 (CH), 114.7 (CH), 104.7 (CH), 55.7 (CH_3);

IR (neat) ν = 3003 (w), 1529 (m), 1516 (s), 1458 (m), 1363 (m), 1257 (s), 1234 (m), 1045 (m), 1025 (m), 956 (m) cm^{-1};

LC-MS (MeCN), Rt. 3.63 min, m/z = 251.1 $[M+H]^+$. HR-MS (ESI-TOF) calculated for $C_{16}H_{15}N_2O$ 251.1184, found 251.1192 (Δ = 3.2 ppm);

M.p. 107-108 °C (*i*PrOH) (Literature: 106-107 °C, aq. EtOH) [6].

Ethyl 2-(4-methoxyphenyl)-6-methyl-3-oxo-2,3-dihydropyridazine-4-carboxylate, 20:

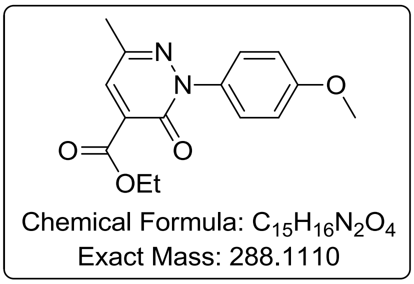

Prepared using general procedure A: Isolated yield; 0.162 g (81%, 0.697 mmol scale);

Yellow crystals; Rf: 0.24 (1/1, EtOAc/hexanes);

^{1}H NMR (400 MHz, $CDCl_3$) δ/ppm 7.65 (s, 1H), 7.52 – 7.45 (m, 2H), 6.99 – 6.92 (m, 2H), 4.40 (q, J = 7.1 Hz, 2H), 3.83 (s, 3H), 2.42 (s, 3H), 1.38 (t, J = 7.1 Hz, 3H);

^{13}C NMR (101 MHz, $CDCl_3$) δ/ppm 163.9 (C), 159.5 (C), 156.5 (C), 143.9 (C), 134.74 (CH), 134.66 (C), 131.4 (C), 126.9 (CH), 114.1 (CH), 62.3 (CH_2), 55.7 (CH_3), 21.0 (CH_3), 14.3 (CH_3);

IR (neat) ν = 3022 (w), 2971 (w), 1739 (s, C=O), 1658 (m, C=O of lactam), 1604 (m), 1510 (m), 1314 (m), 1250 (m), 1229 (s), 1150 (m), 1025 (m), 841 (s) cm^{-1};

LC-MS (MeCN), Rt. 2.64 min, m/z = 289.4 $[M+H]^+$. HR-MS (ESI-TOF) calculated for $C_{15}H_{17}N_2O_4$ 289.1188, found 289.1198 (Δ = 3.5 ppm);

Elemental analysis: calculated for $C_{15}H_{16}N_2O_4$ C: 62.49%, H: 5.59%, N: 9.72%; measured C: 62.63% (Δ = 0.14), H: 5.59% (Δ = 0.00), N: 9.73% (Δ = 0.01);

M.p. 80-81 °C (*i*PrOH).

4-Methoxy-*N*-phenylaniline, 21:

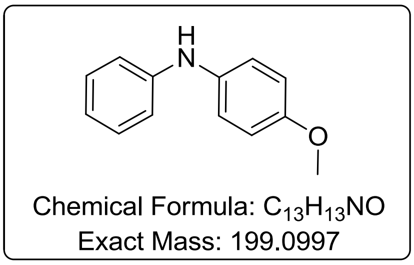

Consistent with published data [7].

Prepared using general procedure A: Isolated yield; 0.125 g (90%, 0.69 mmol scale);

Colourless crystals; Rf: 0.36 (8/2, EtOAc/hexanes);

^{1}H NMR (400 MHz, $CDCl_3$) δ/ppm 7.28 – 7.22 (m, 2H), 7.14 – 7.08 (m, 2H), 6.98 – 6.83 (m, 5H), 5.54 (s, br, 1H), 3.83 (s, 3H);

^{13}C NMR (101 MHz, $CDCl_3$) δ/ppm 155.4 (C), 145.3 (C), 135.9 (C), 129.4 (CH), 122.4 (CH), 119.7 (CH), 115.8 (CH), 114.8 (CH), 55.7 (CH_3);

IR (neat) ν = 3387 (m), 3010 (w), 2958 (w), 2839 (w), 1595 (m), 1507 (s), 1499 (s), 1462 (w), 1443 (m), 1316 (m), 1298 (m), 1248 (s), 1236 (s), 1182 (m), 1169 (m), 1033 (w), 812 (m), 750 (s), 694 (s) cm^{-1};

LC-MS (MeCN), Rt. 3.15 min, m/z = 200.6 $[M+H]^+$. HR-MS (ESI-TOF) calculated for $C_{13}H_{14}NO$ 200.1075, found 200.1072 (Δ = 1.5 ppm);

M.p. 102-104 °C (MeCN) (Literature: 101-103 °C, no solvent reported) [7].

Diphenylamine, 22:

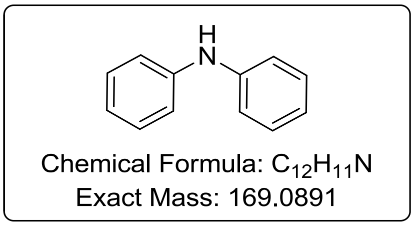

Consistent with published data [8].

Prepared using general procedure A: Isolated yield; 0.108 g (92%, 0.69 mmol scale);

Colourless crystals; Rf: 0.47 (8/2, EtOAc/hexanes);

^{1}H NMR (400 MHz, $CDCl_3$) δ/ppm 7.33 – 7.26 (m, 4H), 7.13 – 7.07 (m, 4H), 6.96 (tt, *J* = 7.3, 1.1 Hz, 2H), 5.82 (s, br, 1H);

^{13}C NMR (101 MHz, $CDCl_3$) δ/ppm 143.2 (C), 129.5 (CH), 121.2 (CH), 118.0 (CH);

IR (neat) ν = 3408 (w), 3384 (m), 3042 (w), 1739 (w, br), 1596 (s), 1519 s), 1496 (s), 1459 (m), 1419 (m), 1319 (m), 1173 (m), 749 (s), 744 (s), 690 (s) cm^{-1};

LC-MS (MeCN), Rt. 3.22 min, m/z = 170.1 $[M+H]^+$. HR-MS (ESI-TOF) calculated for $C_{12}H_{12}N$ 170.0970, found 170.0966 (Δ = 2.4 ppm);

M.p. 53-54 °C (95% EtOH) (Literature: 54-55 °C, no solvent reported) [8].

5-Bromo-*N*-phenylpyridin-3-amine, 23:

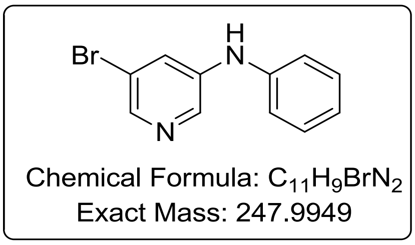

Prepared using general procedure A: Isolated yield; 0.085 g (50%, 0.69 mmol scale);

Colourless crystals; Rf: 0.20 (8/2, EtOAc/hexanes);

^{1}H NMR (400 MHz, $CDCl_3$) δ/ppm 8.24 (d, *J* = 2.5 Hz, 1H), 8.16 (d, *J* = 2.0 Hz, 1H), 7.52 (t, *J* = 2.2 Hz, 1H), 7.38 – 7.30 (m, 2H), 7.15 – 7.03 (m, 3H), 6.02 (s, br, 1H);

^{13}C NMR (101 MHz, $CDCl_3$) δ/ppm 141.9 (CH), 141.5 (C), 140.7 (C), 137.5 (CH), 130.0 (CH), 124.6 (CH), 123.4 (CH), 121.0 (C), 119.7 (CH);

IR (neat) ν = 3257 (w), 3083 (w), 3047 (w), 2992 (w), 2365 (w), 1739 (w, br), 1614 (w), 1578 (s), 1570 (s), 1497 (s), 1444 (s), 1343 (m), 1331 (m), 1219 (m), 1096 (m), 1005 (m), 854 (s), 750 (s), 693 (s) cm^{-1};

LC-MS (MeCN), Rt. 3.09 min, m/z = 249.0 $[M+H]^+$. HR-MS (ESI-TOF) calculated for $C_{11}H_{10}N_2Br$ 249.0027, found 249.0023 (Δ = 1.6 ppm);

M.p. 160 °C (DCM).

4-Chloro-*N*-phenylaniline, 24:

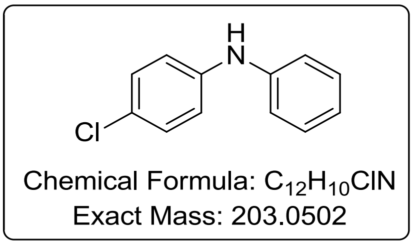

Consistent with published data [7].

Prepared using general procedure A: Isolated yield; 0.201 g (71%, 1.39 mmol scale);

Yellow oil; Rf: 0.44 (8/2, EtOAc/hexanes);

^{1}H NMR (400 MHz, $CDCl_3$) δ/ppm 7.32 – 7.25 (m, 3H), 7.24 – 7.19 (m, 2H), 7.08 – 7.03 (m, 2H), 7.02 – 6.93 (m, 3H), 5.79 (s, br, 1H);

^{13}C NMR (101 MHz, $CDCl_3$) δ/ppm 142.7 (C), 141.9 (C), 129.6 (CH), 129.4 (CH), 125.7 (C), 121.7 (CH), 119.0 (CH), 118.3 (CH).

IR (neat) ν = 3400 (w, br), 3062 (w), 3029 (w), 1588 (s), 1501 (s), 1499 (s), 1496 (s), 1486 (s), 1310 (s), 1173 (m), 1091 (m), 816 (m), 748 (s), 693 (s) cm^{-1};

LC-MS (MeCN), Rt. 3.29 min, m/z = 204.11 $[M+H]^+$. HR-MS (ESI-TOF) calculated for $C_{12}H_{11}NCl$ 204.0580, found 204.0579 (Δ = 0.5 ppm).

(*Rac*)-methyl 3-(4-hydroxyphenyl)-2-(phenylamino)propanoate, 25:

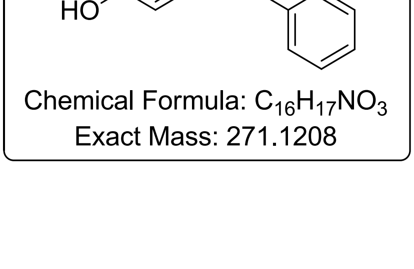

Prepared using general procedure A: Isolated yield; 0.049 g (26%, 0.69 mmol scale);

Colourless crystals; Rf: 0.12 (8/2, EtOAc/hexanes);

^{1}H NMR (400 MHz, $CDCl_3$) δ/ppm 7.18 (dd, *J* = 7.3, Hz, 2H), 7.02 (d, *J* = 8.4 Hz, 2H), 6.76 (t, *J* = 7.3 Hz, 1H), 6.74 (d, *J* = 8,4 Hz, 2H), 6.63 (d, *J* = 7.7 Hz, 2H), 5.08 (s, 1H), 4.33 (t, *J* = 6.1 Hz, 1H), 4.15 (s, br, 1H), 3.68 (s, 3H), 3.14 – 3.00 (m, 2H);

^{13}C NMR (101 MHz, $CDCl_3$) δ/ppm 174.0 (C), 154.8 (C), 146.4 (C), 130.6 (CH), 129.5 (CH), 128.3 (C), 118.7 (CH), 115.6 (CH), 113.8 (CH), 58.0 (CH), 52.3 (CH_3), 37.9 (CH_2);

IR (neat) ν = 3391 (m, br), 3028 (w). 2954 (w), 1727 (m, C=O), 1603 (s), 1515 (s), 1437 (w), 1221 (m, br) cm^{-1};

LC-MS (MeCN), Rt. 2.61 min m/z = 272.4 $[M+H]^+$. HR-MS (ESI-TOF) calculated for $C_{16}H_{18}NO_3$ 272.1287, found 272.1281 (Δ = 2.2 ppm).

(*Rac*)-methyl 3-(4-phenoxyphenyl)-2-(phenylamino)propanoate, 26:

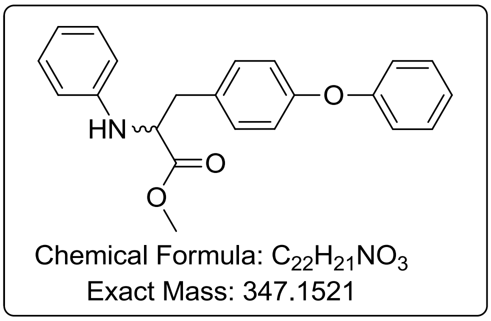

The results from the 2D NMR spectra (2D-NOESY) are consistent with the second alkylation on the phenol rather than the secondary amine. The fact that correlation is present with the ortho-protons on the phenol attached to the amine and the methyl and methylene protons but no other correlation to other aromatic protons with the same methane and methylene protons, indicates that the other phenyl ring is not in the same environment. Unfortunately no correlation with the ortho-protons of the phenyl rings separated by the ether bond is present, but this could be due to the conformation of the molecule with the phenyl rings perpendicular to each other.

Prepared using general procedure A: Isolated yield; 8 mg (3%, 0.69 mmol scale);

Colourless crystals; Rf: 0.40 (8/2, EtOAc/hexanes);

^{1}H NMR (700 MHz, $CDCl_3$) δ/ppm 7.35 – 7.31 (m, 2H), 7.20 – 7.16 (m, 2H), 7.14 – 7.08 (m, 3H), 7.00 – 6.97 (m, 2H), 6.95 – 6.92 (m, 2H), 6.76 (tt, *J* = 7.3, 1.1 Hz, 1H), 6.64 – 6.60 (m, 2H), 4.35 (t, *J* = 6.2 Hz, 1H), 3.69 (s, 3H), 3.18 – 3.06 (m, 2H);

^{13}C NMR (176 MHz, $CDCl_3$) δ/ppm 173.6 (C), 157.3 (C), 156.5 (C), 146.3 (C), 131.2 (C), 130.7 (CH), 129.9 (CH), 129.5 (CH), 123.4 (CH), 119.04 (CH), 119.01 (CH), 118.8 (CH), 113.9 (CH), 58.1 (CH), 52.3 (CH_3), 38.1 (CH_2);

IR (neat) ν = 3404 (w), 3030 (w), 2954 (w), 1739 (m, C=O), 1603 (m), 1591 (m), 1506 (s), 1489 (s), 1239 (s) cm^{-1};

LC-MS (MeCN), Rt. 3.67 min, m/z = 348.2 $[M+H]^+$. HR-MS (ESI-TOF) calculated for $C_{22}H_{22}NO_3$ 348.1600, found 348.1609 (Δ = 2.6 ppm).

Methyl 4-methyl-2-(phenylamino)pentanoate, 27:

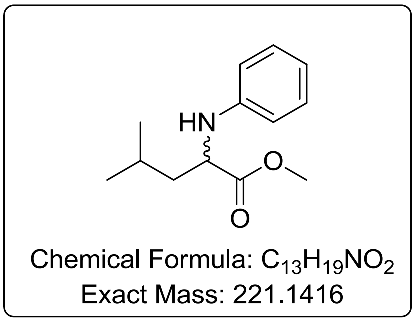

Prepared using general procedure A: Isolated yield; 0.153 g (60%, 0.69 mmol scale);

Amorphous solid; Rf: 0.52 (8/2, EtOAc/hexanes);

^{1}H NMR (400 MHz, $CDCl_3$) δ/ppm 7.22 – 7.14 (m, 2H), 6.75 (tt, *J* = 7.4, 1.1 Hz, 1H), 6.65 – 6.60 (m, 2H), 4.11 (dd, *J* = 7.8, 6.6 Hz, 1H), 4.00 (s, br, 1H), 3.71 (s, 3H), 1.81 (dh, *J* = 13.3, 6.6 Hz, 1H), 1.72 – 1.59 (m, 2H), 1.00 (d, *J* = 6.6 Hz, 3H), 0.95 (d, *J* = 6.6 Hz, 3H);

^{13}C NMR (101 MHz, $CDCl_3$) δ/ppm 175.3 (C), 147.1 (C), 129.5 (CH), 118.5 (CH), 113.5 (CH), 55.2 (CH), 52.2 (CH_3), 42.5 (CH_2), 25.0 (CH), 22.9 (CH_3), 22.32 (CH_3);

IR (neat) ν = 3384 (w, br), 3028 (w), 2955 (m), 2870 (w), 1734 (s), 1602 (s), 1507 (m), 1433 (w), 1198 (m), 1155 (s), 748 (s), 691 (s) cm^{-1};

LC-MS (MeCN), Rt. 3.39 min, m/z = 222.2 $[M+H]^+$. HR-MS (ESI-TOF) calculated for $C_{13}H_{20}NO_2$ 222.1494, found 222.1496 (Δ = 0.9 ppm).

1-(3,4-Dimethoxyphenyl)-3,4-dimethyl-1*H*-1,2,4-triazol-5(4*H*)-one, 28:

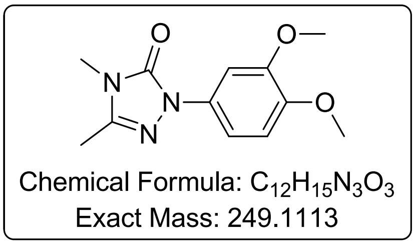

Prepared using general procedure A: Isolated yield; 0.045g (26%, 0.702 mmol scale);

Off-white crystals (recrystallised using *i*PrOH); Rf: 0.12 (8/2, EtOAc/hexanes);

^{1}H NMR (600 MHz, $CDCl_3$) δ/ppm 7.56 (d, *J* = 2.4 Hz, 1H), 7.45 (dd, *J* = 8.8, 2.4 Hz, 1H), 6.89 (d, *J* = 8.8 Hz, 1H), 3.93 (s, 3H), 3.89 (s, 3H), 3.29 (s, 3H), 2.30 (s, 3H);

^{13}C NMR (151 MHz, $CDCl_3$) δ/ppm 152.3 (C), 149.0 (C), 146.5 (C), 143.8 (C), 131.6 (C), 111.2 (CH), 110.7 (CH), 103.2 (CH), 56.1 (CH_3), 55.9 (CH_3), 27.4 (CH_3), 11.8 (CH_3);

IR (neat) ν = 2943 (w), 2842 (w), 1701 (s), 1604 (w). 1590 (w), 1515 (s), 1466 (m), 1250 (s), 1220 (m), 1027 (m) cm^{-1};

LC-MS (MeCN), Rt. 2.48 min, m/z = 250.1 $[M+H]^+$. HR-MS (ESI-TOF) calculated for $C_{12}H_{16}N_3O_3$ 250.1192, found 250.1196 (Δ = 1.6 ppm);

Elemental analysis: calculated for $C_{12}H_{15}N_3O_3$ C: 57.82%, H: 6.07%, N: 16.86%; measured C: 57.60% (Δ = 0.22), H: 6.08% (Δ = 0.01), N: 16.51% (Δ = 0.35);

M.p. 145-146 °C (*i*PrOH).

1,3-Diphenyl-1*H*-pyrazole, 29:

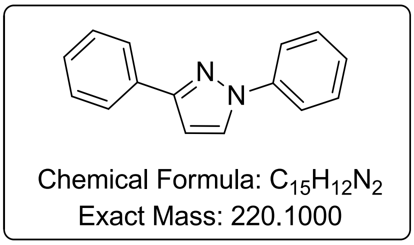

Consistent with published data [5,9].

Prepared using general procedure A: Isolated yield; 0.249 g (81%, 0.70 mmol scale);

Colourless crystals (recrystallised using *i*PrOH); Rf: 0.40 (8/2, EtOAc/hexanes);

^{1}H NMR (700 MHz, $CDCl_3$) 7.99 – 7.95 (m, 1H), 7.93 (dd, *J* = 7.8, 1.5 Hz, 2H), 7.80 – 7.76 (m, 2H), 7.50 – 7.46 (m, 2H), 7.44 (t, *J* = 7.6 Hz, 2H), 7.37 – 7.33 (m, 1H), 7.32 – 7.28 (m, 1H), 6.80 – 6.77 (m, 1H);

^{13}C NMR (176 MHz, $CDCl_3$) δ/ppm 153.1 (C), 140.4 (C), 133.2 (C), 129.6 (CH), 128.8 (CH), 128.2 (CH), 128.1 (CH), 126.5 (CH), 126.0 (CH), 119.2 (CH), 105.2 (CH);

IR (neat) ν = 3137 (w), 3065 (w), 1599 (m), 1526 (m), 1506 (m), 1457 (m), 1360 (m), 1303 (w), 1265 (m), 1045 (m), 954 (m), 940 (m) cm^{-1};

LC-MS (MeCN), Rt. 3.53 min, m/z = 221.2 $[M+H]^+$. HR-MS (ESI-TOF) calculated for $C_{15}H_{13}N_2$ 221.1079, found 221.1090 (Δ = 5.0 ppm);

M.p. 84-85 °C (*i*PrOH) (Literature: 83-84 °C, no solvent reported) [9].

1-(3-(Methylthio)phenyl)-3-phenyl-1*H*-pyrazole, 30:

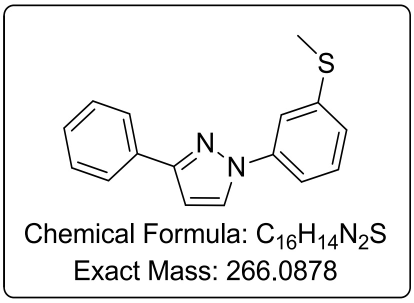

Prepared using general procedure A: Isolated yield; 0.286 g (77%, 1.4 mmol scale);

Colourless oil; Rf: 0.50 (8/2, EtOAc/hexanes);

^{1}H NMR (600 MHz, $CDCl_3$) δ/ppm 7.95 (d, *J* = 1.4 Hz, 1H), 7.94 – 7.92 (m, 2H), 7.73 (t, *J* = 2.2 Hz, 1H), 7.49 (ddd, *J* = 8.2, 2.2, 0.9 Hz, 1H), 7.45 (dd, *J* = 8.2, 7.1 Hz, 2H), 7.36 (t, *J* = 7.7 Hz, 2H), 7.19 – 7.15 (m, 1H), 6.78 (d, *J* = 2.4 Hz, 1H), 2.56 (s, 3H);

^{13}C NMR (151 MHz, $CDCl_3$) δ/ppm 153.1 (C), 140.7 (C), 140.5 (C), 133.1 (C), 129.7 (CH), 128.8 (CH), 128.2 (CH), 128.2 (CH), 126.0 (CH), 124.2 (CH), 117.0 (CH), 115.5 (CH), 105.3 (CH), 15.8 (CH_3);

IR (neat) ν = 3062 (w), 2920 (w), 1591 (s), 1583 (s), 1530 (w), 1502 (s), 1479 (m), 1454 (s), 1360 (s), 1045 (s), 963 (m), 945 (m) cm^{-1};

LC-MS (MeCN), Rt. 4.409 min, m/z = 267.1 $[M+H]^+$. HR-MS (ESI-TOF) calculated for 267.0956 $C_{16}H_{15}N_2S$, found 267.0966 (Δ = 3.7 ppm).

1-(2-Methoxyphenyl)-3-phenyl-1*H*-pyrazole, 31:

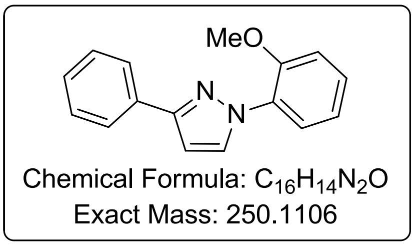

Consistent with published data[5]

Prepared using general procedure A: Isolated yield; 0.227 g (65%, 1.4 mmol scale);

Colourless oil; Rf: 0.43 (8/2, EtOAc/hexanes);

^{1}H NMR (400 MHz, $CDCl_3$) δ/ppm 8.09 (d, *J* = 2.5 Hz, 1H), 7.94 – 7.90 (m, 2H), 7.87 (dd, *J* = 7.9, 1.7 Hz, 1H), 7.44 – 7.40 (m, 2H), 7.35 – 7.29 (m, 2H), 7.09 (td, *J* = 7.7, 1.3 Hz, 1H), 7.06 (dd, *J* = 8.3, 1.3 Hz, 1H), 6.74 (d, *J* = 2.5 Hz, 1H), 3.91 (s, 3H);

^{13}C NMR (151 MHz, $CDCl_3$) δ/ppm 151.8 (C), 151.2 (C), 133.3 (C), 133.0 (CH), 129.8 (C), 128.5 (CH), 127.8 (CH), 127.8 (CH), 125.9 (CH), 125.2 (CH), 121.3 (CH), 112.3 (CH), 103.7 (CH), 56.0 (CH_3);

IR (neat) ν = 3060 (w), 2940 (w), 2839 (w), 1597 (m), 1530 (m), 1508 (s), 1455 (s), 1286 (m), 1259 (m), 1242 (s), 1127 (m), 1022 (s), 954 (m), 942 (m) cm^{-1};

LC-MS (MeCN), Rt. 3.53 min, m/z = 251.2 $[M+H]^+$. HR-MS (ESI-TOF) calculated for 251.14182 $C_{16}H_{15}N_2O$, found 251.1182 (Δ = 0.0 ppm).

1-(3-Methoxyphenyl)-3-phenyl-1*H*-pyrazole, 32:

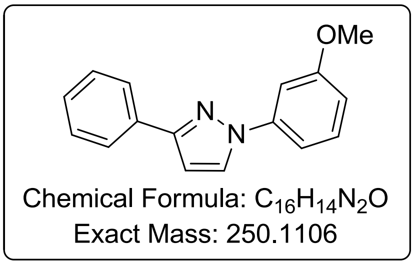

Consistent with published data [5].

Prepared using general procedure A: Isolated yield; 0.287 g (82%, 1.4 mmol scale);

Colourless oil; Rf: 0.36 (8/2, EtOAc/hexanes);

^1H NMR (400 MHz, $CDCl_3$) δ/ppm 8.01 – 7.97 (m, 2H), 7.96 (d, *J* = 2.5 Hz, 1H), 7.52 – 7.44 (m, 3H), 7.43 – 7.32 (m, 3H), 6.88 (ddd, *J* = 8.0, 2.5, 1.2 Hz, 1H), 6.80 (d, *J* = 2.5 Hz, 1H), 3.92 (s, 3H);

^{13}C NMR (151 MHz, $CDCl_3$) δ/ppm 160.6 (C), 152.9 (C), 141.4 (C), 133.2 (C), 130.2 (CH), 128.7 (CH), 128.2 (CH), 128.1 (CH), 125.9 (CH), 112.1 (CH), 111.1 (CH), 105.2 (CH), 105.1 (CH), 55.6 (CH_3);

IR (neat) ν = 3065 (w), 2961 (w), 1606 (s), 1593 (s), 1530 (m), 1504 (s), 1362 (m), 1246 (m), 1217 (s), 1170 (m), 1045 (s), 966 (m) cm^{-1};

LC-MS (MeCN), Rt. 3.35 min, m/z = 251.1 $[M+H]^+$. HR-MS (ESI-TOF) calculated for $C_{16}H_{15}N_2O$ 251.1184, found 251.1194 (Δ = 4.0 ppm).

4-(3-Phenyl-1*H*-pyrazol-1-yl)benzonitrile, 33:

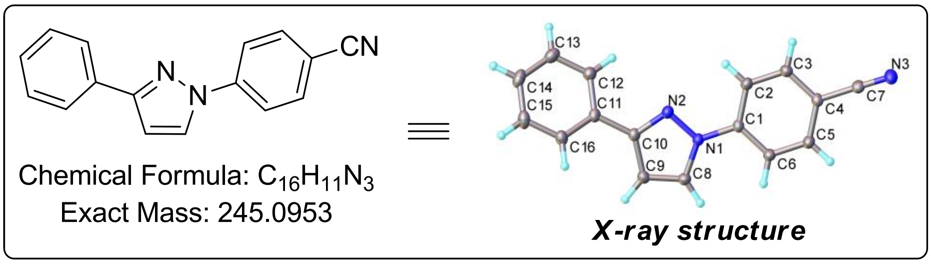

Prepared using general procedure A: Isolated yield; 0.264 g (76%, 1.4 mmol scale);

Colourless crystals (recrystallised using *i*PrOH); Rf: 0.26 (8/2, EtOAc/hexanes);

^{1}H NMR (600 MHz, $CDCl_3$) 8.02 (d, J = 2.6 Hz, 1H), 7.94 – 7.88 (m, 4H), 7.78 – 7.73 (m, 2H), 7.45 (dd, J = 8.3, 7.0 Hz, 2H), 7.41 – 7.36 (m, 1H), 6.84 (d, J = 2.6 Hz, 1H);

^{13}C NMR (151 MHz, $CDCl_3$) δ/ppm 154.2 (C), 142.9 (C), 133.6 (CH), 132.3 (C), 128.8 (CH), 128.6 (CH), 128.0 (CH), 126.0 (CH), 118.7 (CH), 118.5 (CN), 109.3 (C), 106.5 (CH);

IR (neat) ν = 3138 (w), 3123 (w), 2228 (m, CN), 1604 (m), 1533 (m), 1520 (m), 1517 (m), 1457 (m), 1394 (w), 1363 (m), 1183 (m), 953 (m), 939 (m) cm^{-1};

LC-MS (MeCN), Rt. 3.36 min, m/z = 246.1 $[M+H]^{+}$. HR-MS (ESI-TOF) calculated for $C_{16}H_{12}N_3$ 246.1031, found 246.1032 (Δ = 0.4 ppm);

M.p. 126-131 °C (*i*PrOH).

Crystal Data: **33**, $C_{16}H_{11}N_3$ (M =245.28 g/mol): monoclinic, space group $P2_1/c$ (no. 14), a = 10.9809(6) Å, b = 11.0294(6) Å, c = 11.0655(6) Å, β = 113.0712(17)°, V = 1232.98(12) Å^3, Z = 4, T = 120 K, μ(MoKα) = 0.081 mm^{-1}, $Dcalc$ = 1.321 g/cm^3, 26247 reflections measured (5.446° ≤ 2Θ ≤ 60.176°), 3621 unique (R_{int} = 0.0290, R_{sigma} = 0.0186) which were used in all calculations. The final R_1 was 0.0457 (I > 2σ(I)) and wR_2 was 0.1236 (all data).

3-(3-Phenyl-1*H*-pyrazol-1-yl)benzonitrile, 34:

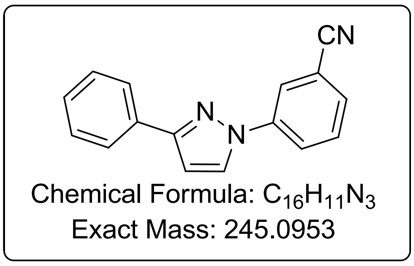

Consistent with published data [5].

Prepared using general procedure A but using 2 equiv. of NEt_3 to aid solubility: Isolated yield; 0.067 g (40%, 0.69 mmol scale);

Colourless oil; Rf: 0.30 (8/2, EtOAc/hexanes);

^{1}H NMR (400 MHz, $CDCl_3$) δ/ppm 8.16 – 8.11 (m, 1H), 8.04 – 7.97 (m, 2H), 7.96 – 7.91 (m, 2H), 7.63 – 7.55 (m, 2H), 7.52 – 7.45 (m, 2H), 7.43 – 7.37 (m, 1H), 6.85 (d, J = 2.6 Hz, 1H);

^{13}C NMR (151 MHz, $CDCl_3$) δ/ppm 153.9 (C), 140.7 (C), 132.5 (C), 130.5 (CH), 129.5 (CH), 128.9 (CH), 128.6 (CH), 127.9 (CH), 126.0 (CH), 122.6 (CH), 122.1 (CH), 118.3 (C), 113.7 (C), 106.3 (CH);

IR (neat) ν = 3148 (w), 3064 (w), 2232 (m, CN), 1604 (s), 1587 (s), 1533 (m), 1506 (s), 1455 (s), 1437 (m), 1398 (m), 1368 (s), 1388 (w), 1051 (m), 967 (m) cm^{-1};

LC-MS (MeCN) Rt. 3.18 min, m/z = 246.4 $[M+H]^+$. HR-MS (ESI-TOF) calculated for $C_{16}H_{15}N_2O$ 246.1031, found 246.1040 (Δ = 3.7 ppm).

2-(3-Phenyl-1*H*-pyrazol-1-yl)benzonitrile: 35;

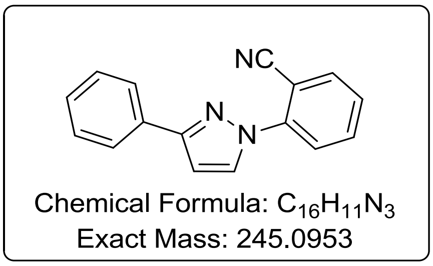

Consistent with published data [10].

Prepared using general procedure A but using 2 equiv. of NEt_3 to aid solubility: Isolated yield; 0.064 g (38%, 0.69 mmol scale);

Colourless oil; Rf: 0.22 (8/2, EtOAc/hexanes);

^{1}H NMR (700 MHz, $CDCl_3$) δ/ppm 8.19 (d, J = 2.6 Hz, 1H), 7.96 – 7.91 (m, 2H), 7.88 (dd, J = 8.3, 1.1 Hz, 1H), 7.79 (dd, J = 7.8, 1.5 Hz, 1H), 7.71 (ddd, J = 8.3, 7.5, 1.5 Hz, 1H), 7.47 – 7.43 (m, 2H), 7.41 (td, J = 7.6, 1.1 Hz, 1H), 7.37 (ddt, J = 8.0, 6.8, 1.3 Hz, 1H), 6.86 (d, J = 2.6 Hz, 1H);

^{13}C NMR (176 MHz, $CDCl_3$) 154.2 (C), 142.0 (C), 134.8 (CH), 134.1 (CH), 132.6 (C), 130.7 (CH), 128.9 (CH), 128.6 (CH), 127.0 (CH), 126.2 (CH), 123.8 (CH), 117.4 (C), 106.2 (CH), 104.9 (C);

IR (neat) ν = 3143 (w), 3065 (w), 2226 (w, CN), 1601 (m), 1580 (m), 1532 (m), 1505 (s), 1455 (s), 1392 (w), 1364 (m), 1310 (w), 1259 (w), 1046 (m), 955 (m), 941 (m) cm^{-1};

LC-MS (MeCN) Rt. 3.11 min, m/z = 246.4 $[M+H]^+$. HR-MS (ESI-TOF) calculated for $C_{16}H_{12}N_3$ 246.1031, found 246.1036 (Δ = 2.0 ppm).

(*E/Z*)-ethyl 2-(1-ethoxyethylidene)hydrazinecarboxylate, 38:

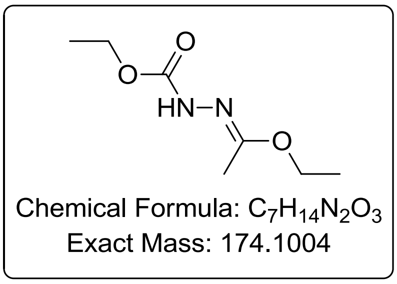

Literature procedure [11].

Ethyl acetimidate hydrochloride (4.96 g, 10 mmol) was dissolved in absolute EtOH (200 mL) and cooled using an ice bath. Ethyl hydrazine carboxylate (4.16 g, 10 mmol) was dissolved in absolute EtOH (80 mL) and added dropwise and reaction left to stir for 6 h at 0 °C. The solvent was evaporated under reduced pressure and the residue was purified using flash chromatography (9:1, DCM/MeOH) to give the pure product as white crystals (5.27 g, 76% yield) as a mixture of two *E*/*Z* isomers (45:55).

Isolated yield: 5.27 g (76%, 10 mmol scale);

White crystals; Rf: 0.72 (1/9, DCM/MeOH);

Isomer 1: ^{1}NMR (700 MHz, $CDCl_3$) δ/ppm 8.06 (s, br, 1H), 4.08 (q, J = 7.0 Hz, 4H), 2.09 (s, 3H), 1.33 (t, J = 7.0 Hz, 6H).

Isomer 2: ^{1}NMR (700 MHz, $CDCl_3$) δ/ppm 6.79 (s, br, 1H), 4.14 (q, J = 7.0 Hz, 4H), 1.94 (s, 3H), 1.27 (t, J = 7.0 Hz, 6H).

IR (neat) ν = 3408 (w), 3274 (w, br), 2986 (w), 2938 (w), 1713 (s), 1657 (s), 1500 (br), 1447 (m), 1379 (m), 1338 (w), 1243 (br), 1047 (s), 1017 (w) cm^{-1};

LC-MS (MeCN), Rt. 2.63 min, m/z = 174.8 $[M+H]^+$. HR-MS ($^+$ESI-TOF) calculated for $C_7H_{14}N_2O_3$ 175.1083, found 175.1078 (Δ = 4.6 ppm).

M.p. 60-65 °C (MeOH) (Literature: 68 °C, Pet. Ether) [12].

3,4-Dimethyl-1*H*-1,2,4-triazol-5(4*H*)-one, 39:

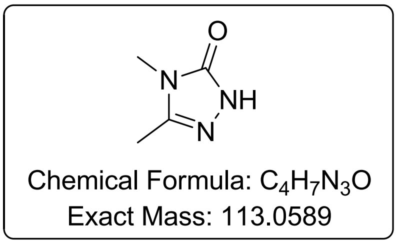

Literature procedure.[13]

Methylamine hydrochloride (2.70 g, 40 mmol) was dissolved in absolute EtOH (200 mL) to which a suspension of sodium ethoxide (2.72 g, 40 mmol) in absolute EtOH (70 mL) was added and reaction was stirred for 5 min at room temperature. A solution of (*E*)-ethyl 2-(1-ethoxyethylidene)hydrazine carboxylate (**38**, 3.48 g, 20 mmol) in absolute EtOH (50 mL) was added dropwise and reaction refluxed for 4 h. The reaction was then cooled to room temperature and filtered over a celite pad. The eluant was dried under reduced pressure and the resultant residue was recrystallised (through a hot filtration) from EtOAc to give the pure product.

Isolated yield: 0.904 g (40%, 20 mmol scale);

White crystals (recrystallised from EtOAc); Rf: 0.31 (1/9, DCM/MeOH);

^{1}H NMR (600 MHz, DMSO-d_6) δ/ppm 11.28 (s, br, 1H), 3.06 (s, 3H), 2.12 (s, 3H);

^{13}C NMR (151 MHz, DMSO-d_6) δ/ppm 155.1 (C), 144.8 (C), 26.3 (CH_3), 11.4 (CH_3);

IR (neat) ν = 3139 (w, br), 3057 (w, br), 3001 (w, br), 2815 (w, br), 1701 (s), 1663 (s), 1590 (m), 1477 (m), 1474 (m), 1437 (m), 1400 (m), 1376 (m), 976 (m), 797 (m). 736 (s), 609 (s) cm^{-1};

LC-MS (MeCN), Rt. 0.77 min, m/z = 114.4 $[M+H]^+$. HR-MS (ESI-TOF) calculated for $C_4H_7N_3O$ 114.0667, found 114.0647 (Δ = 17.5 ppm or -2.0 mDa).

M.p. 146-149 °C (EtOAc) (Literature: 147 °C, no solvent reported) [13].

¹H NMR (700 MHz, $CDCl_3$) (**19**)

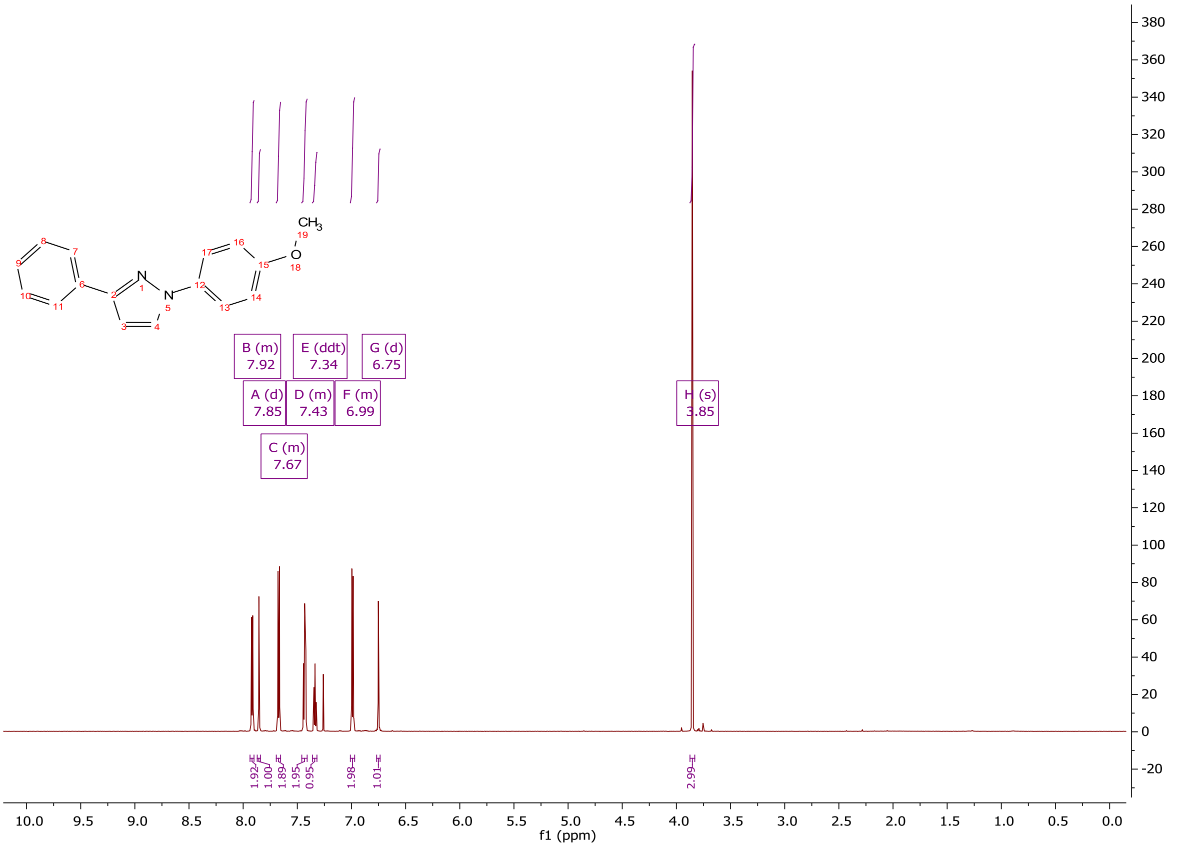

¹³C NMR (176 MHz, $CDCl_3$) (**19**)

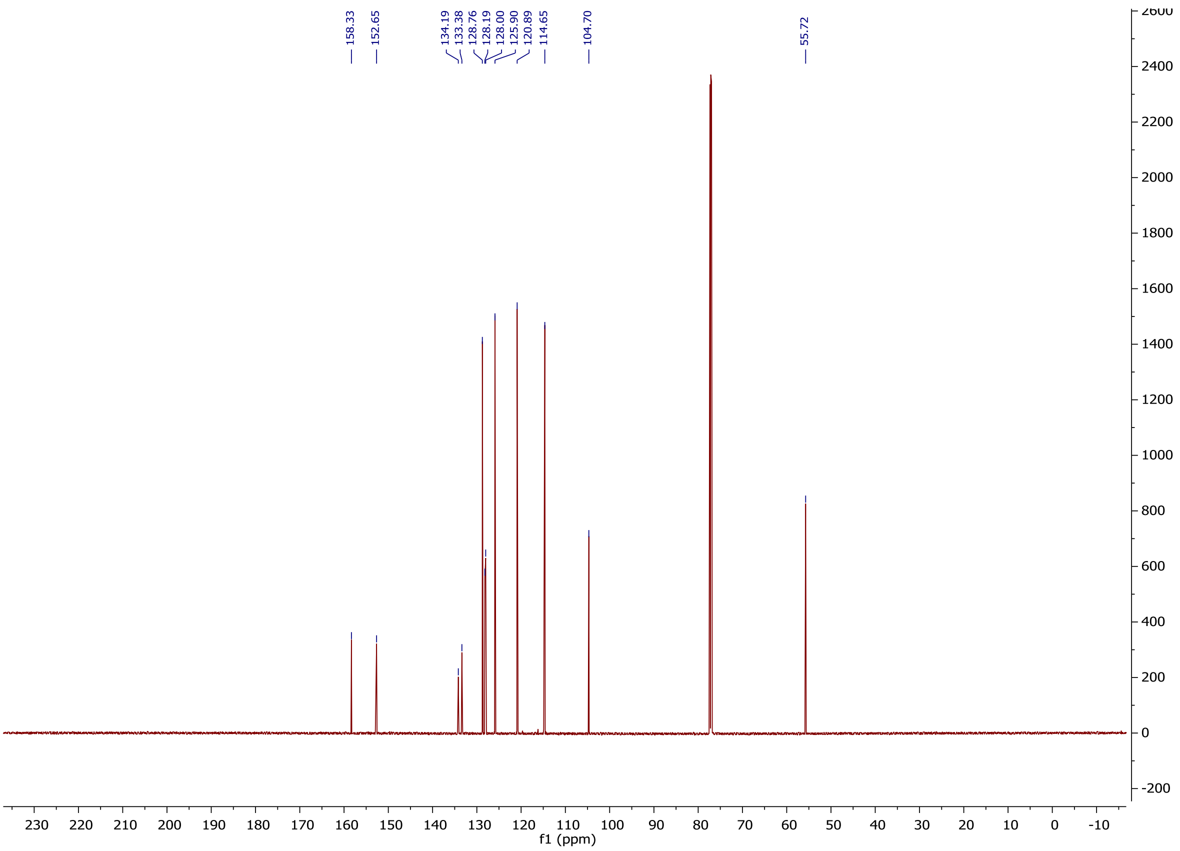

^{1}H *NMR* (400 MHz, $CDCl_3$) (**20**)

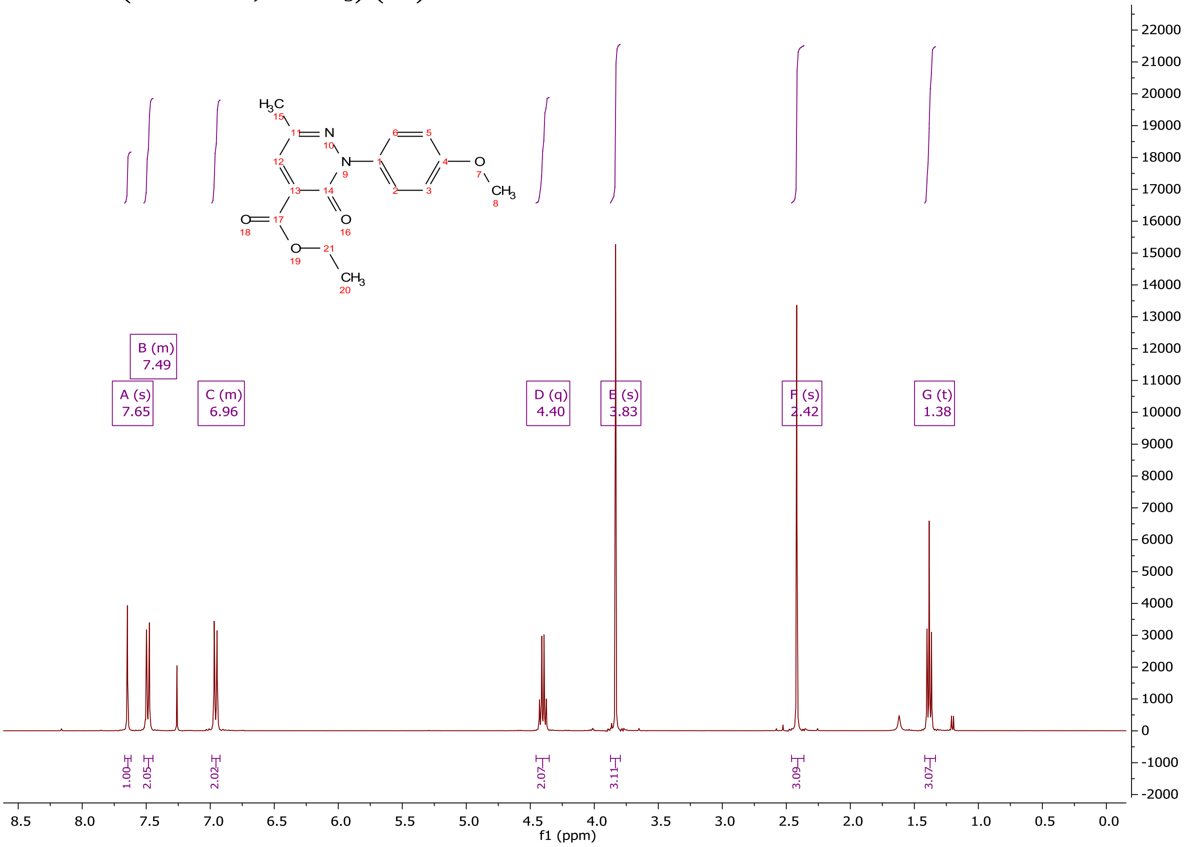

^{13}C *NMR* (101 MHz, $CDCl_3$) (**20**)

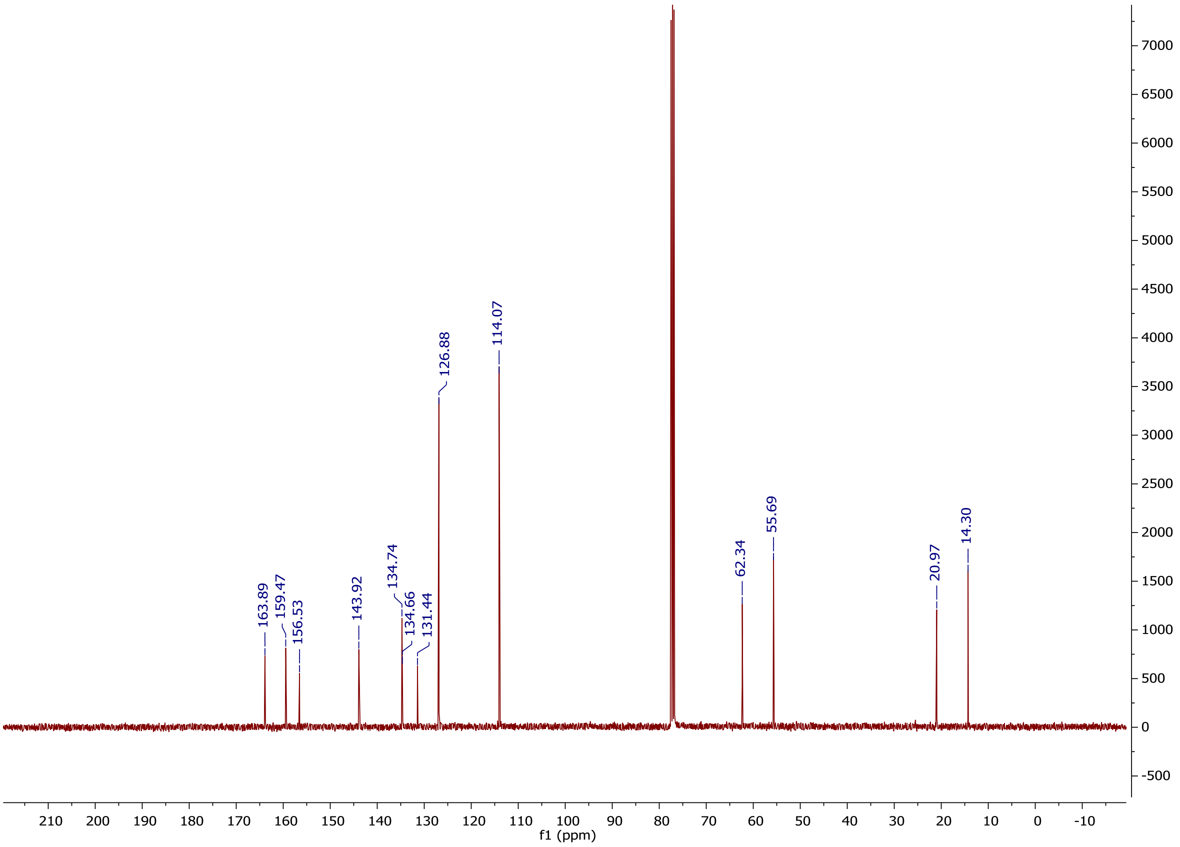

^{1}*H NMR* (400 MHz, $CDCl_3$) (**21**)

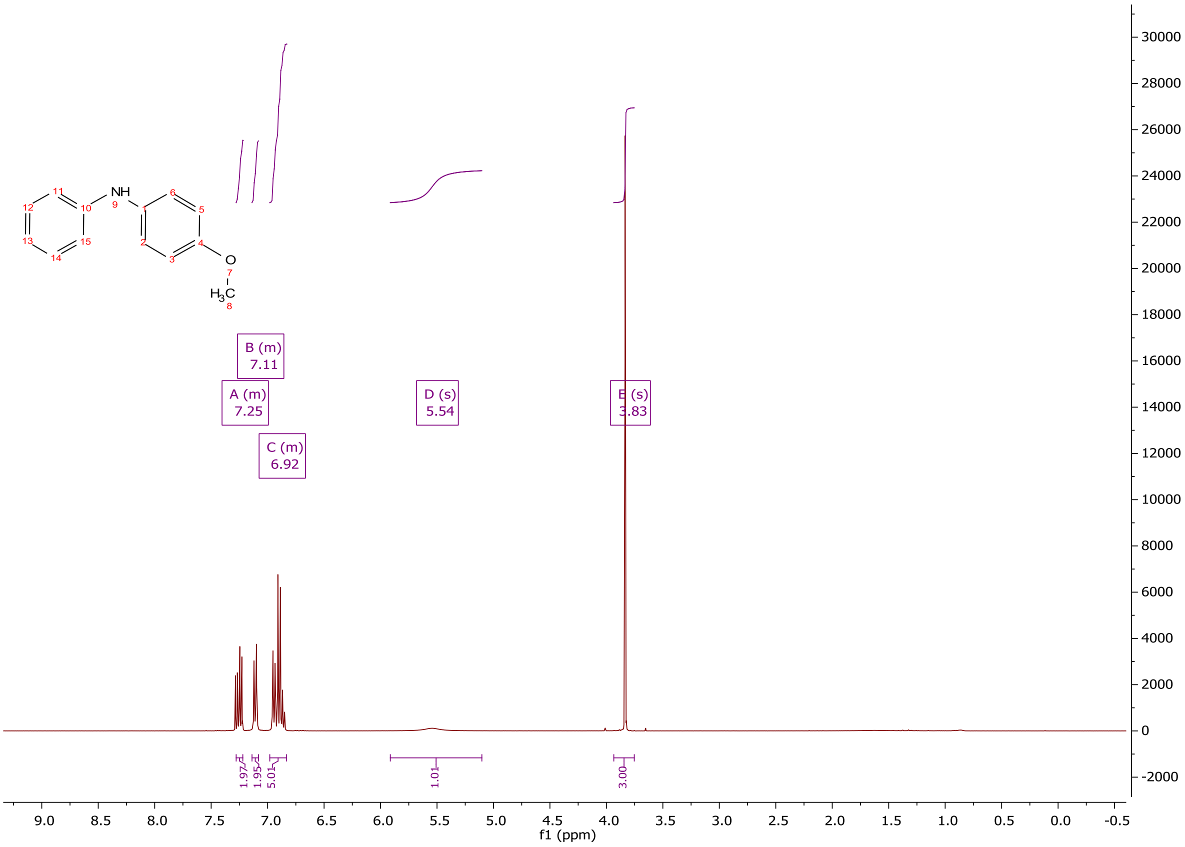

^{13}*C NMR* (101 MHz, $CDCl_3$) (**21**)

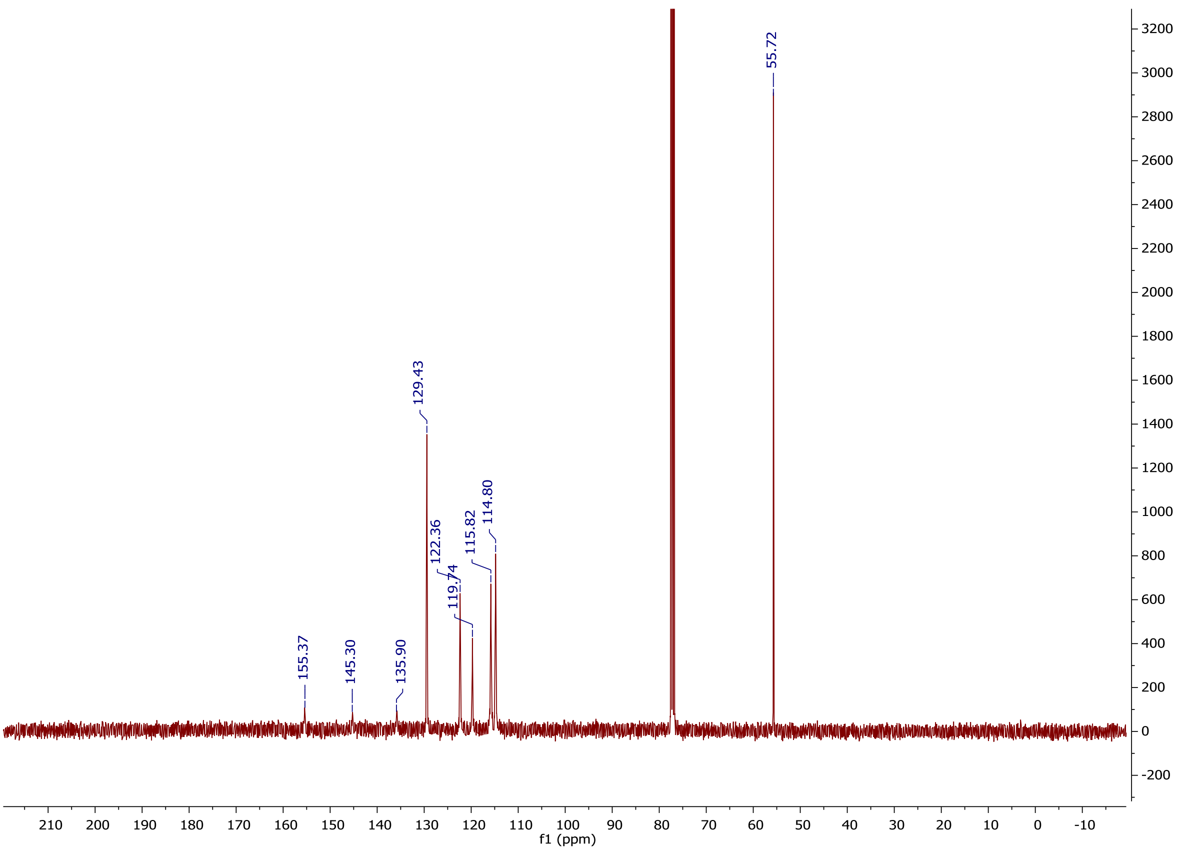

^{1}*H NMR* (400 MHz, $CDCl_3$) (**22**)

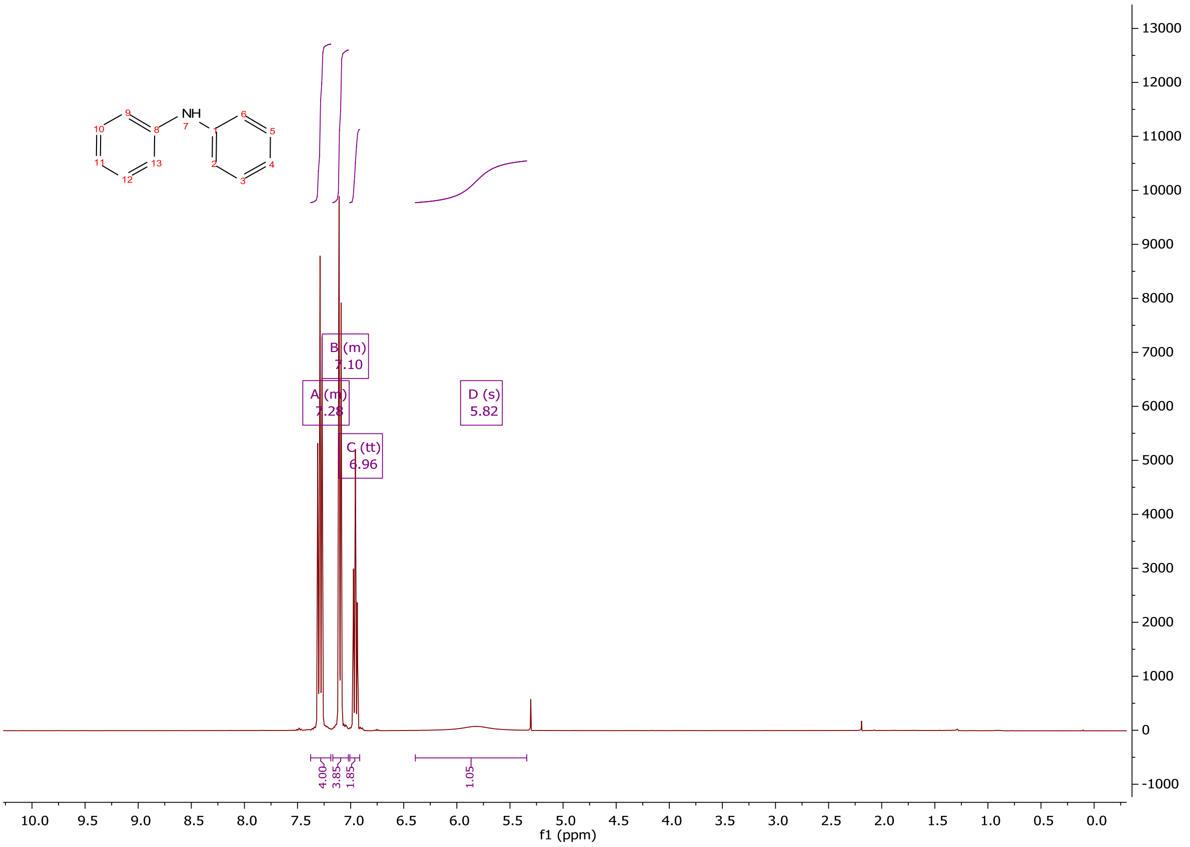

^{13}*C NMR* (101 MHz, $CDCl_3$) (**22**)

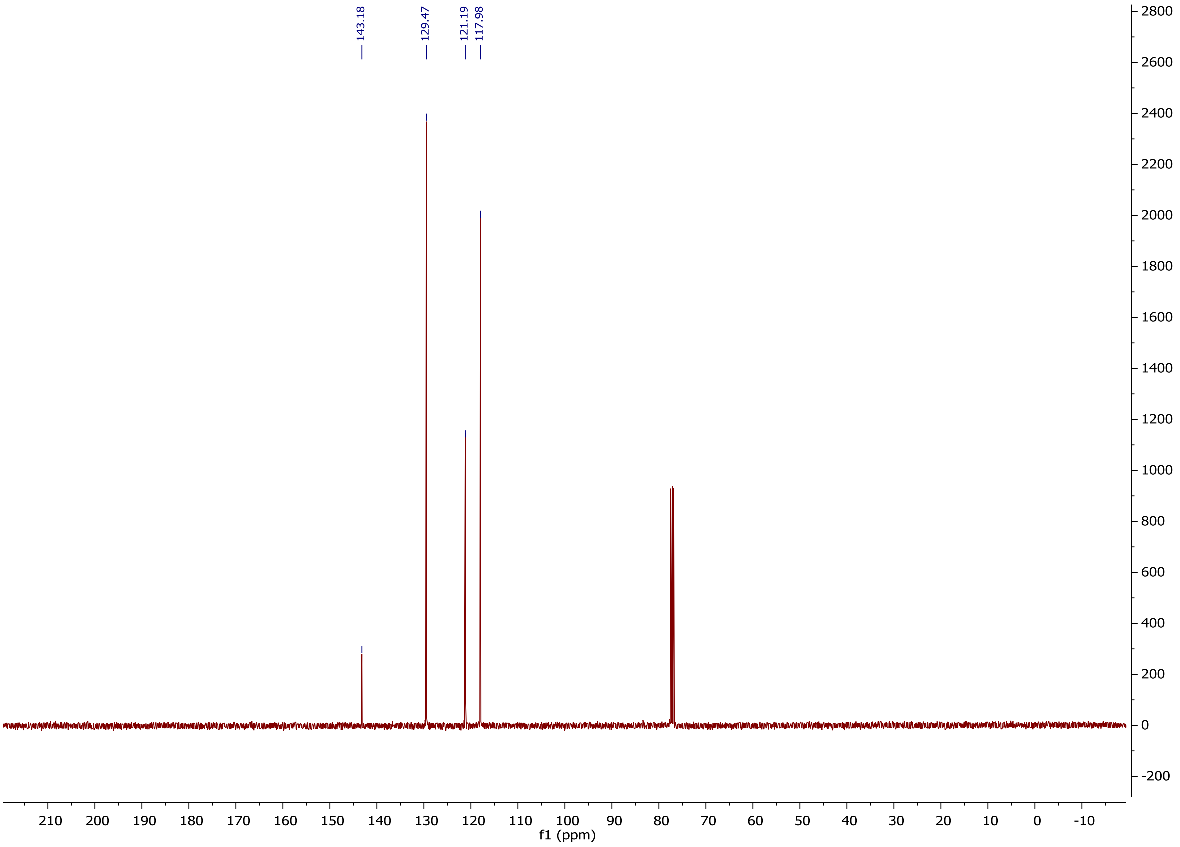

^{1}H NMR (400 MHz, $CDCl_3$) (**23**)

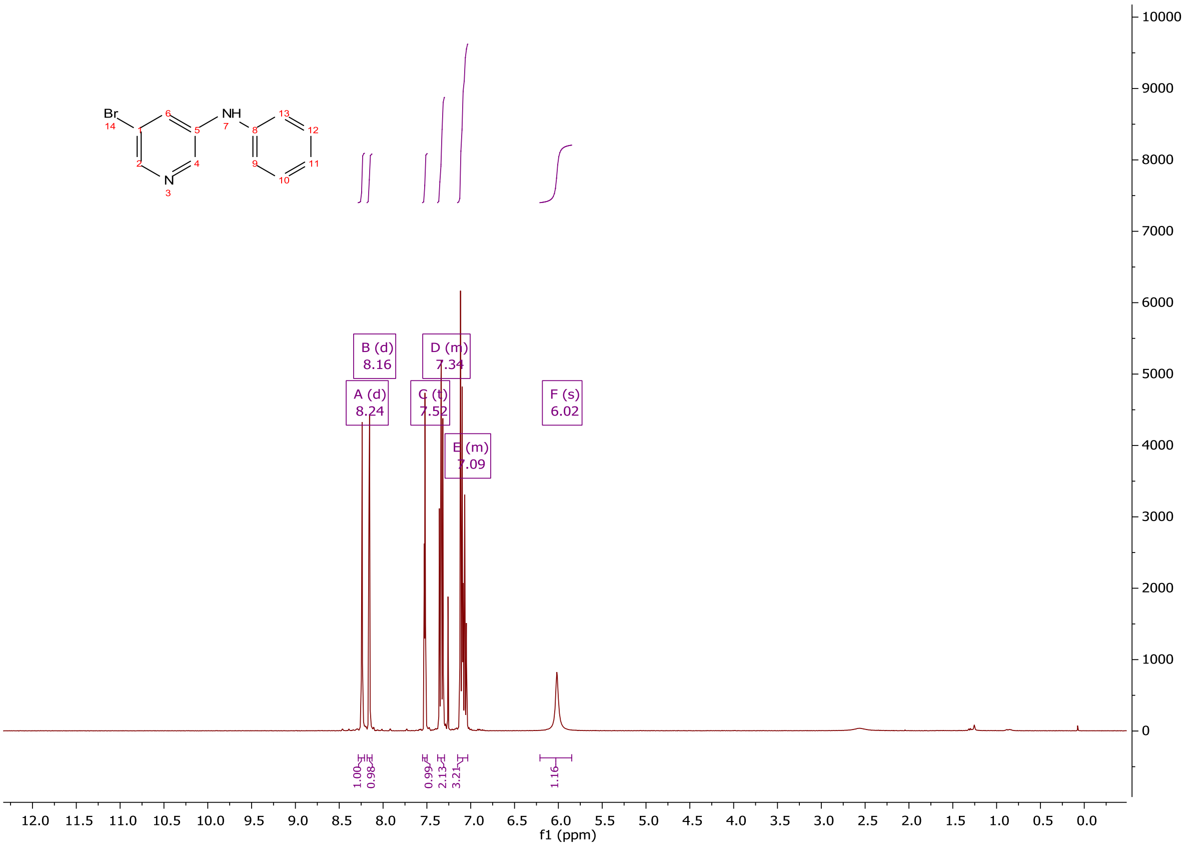

^{13}C NMR (101 MHz, $CDCl_3$) (**23**)

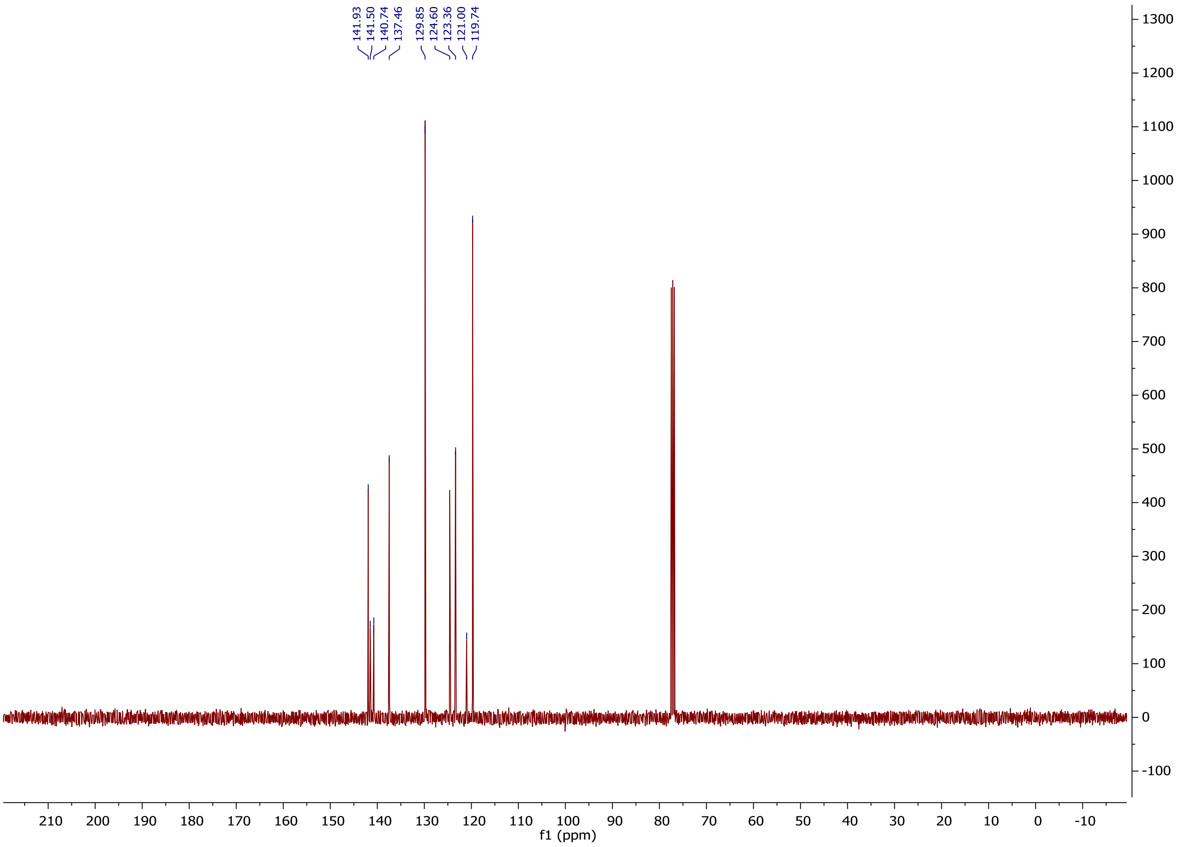

^{1}*H NMR* (400 MHz, $CDCl_3$) (**24**)

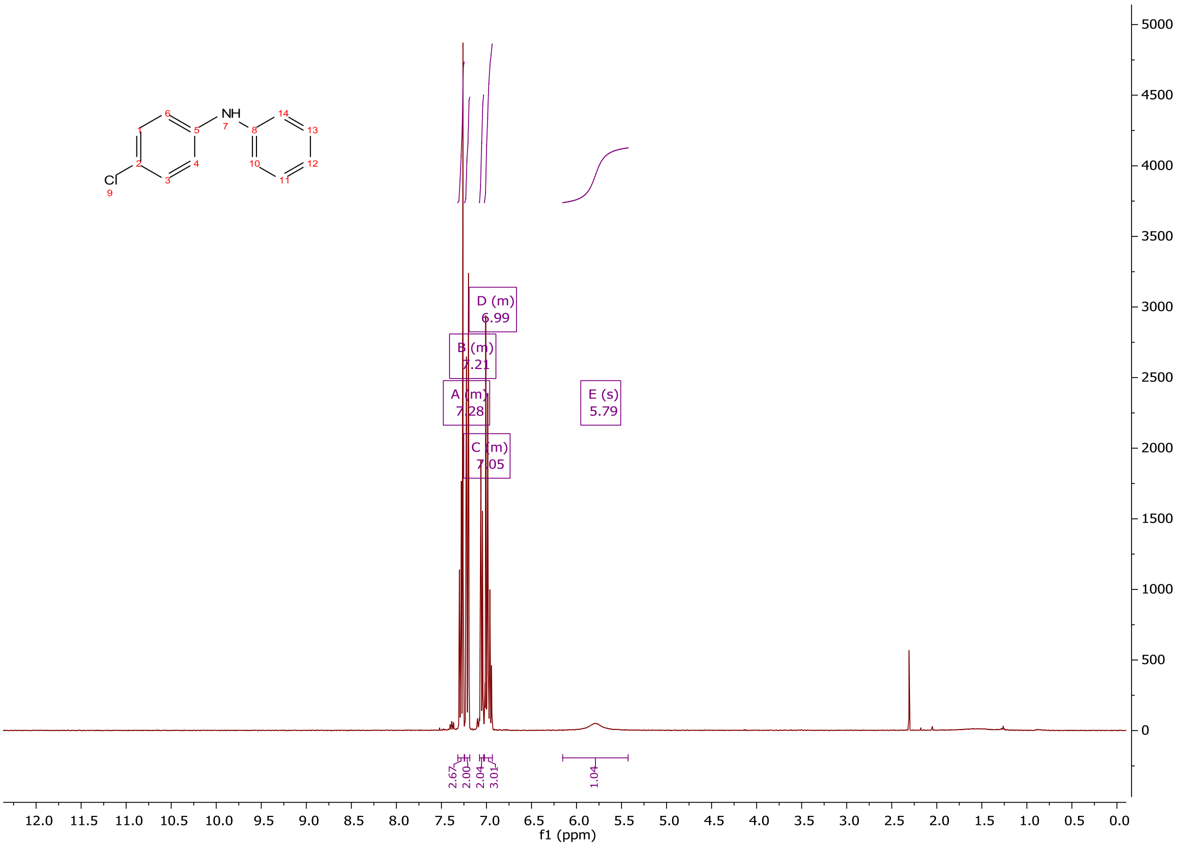

^{13}*C NMR* (101 MHz, $CDCl_3$) (**24**)

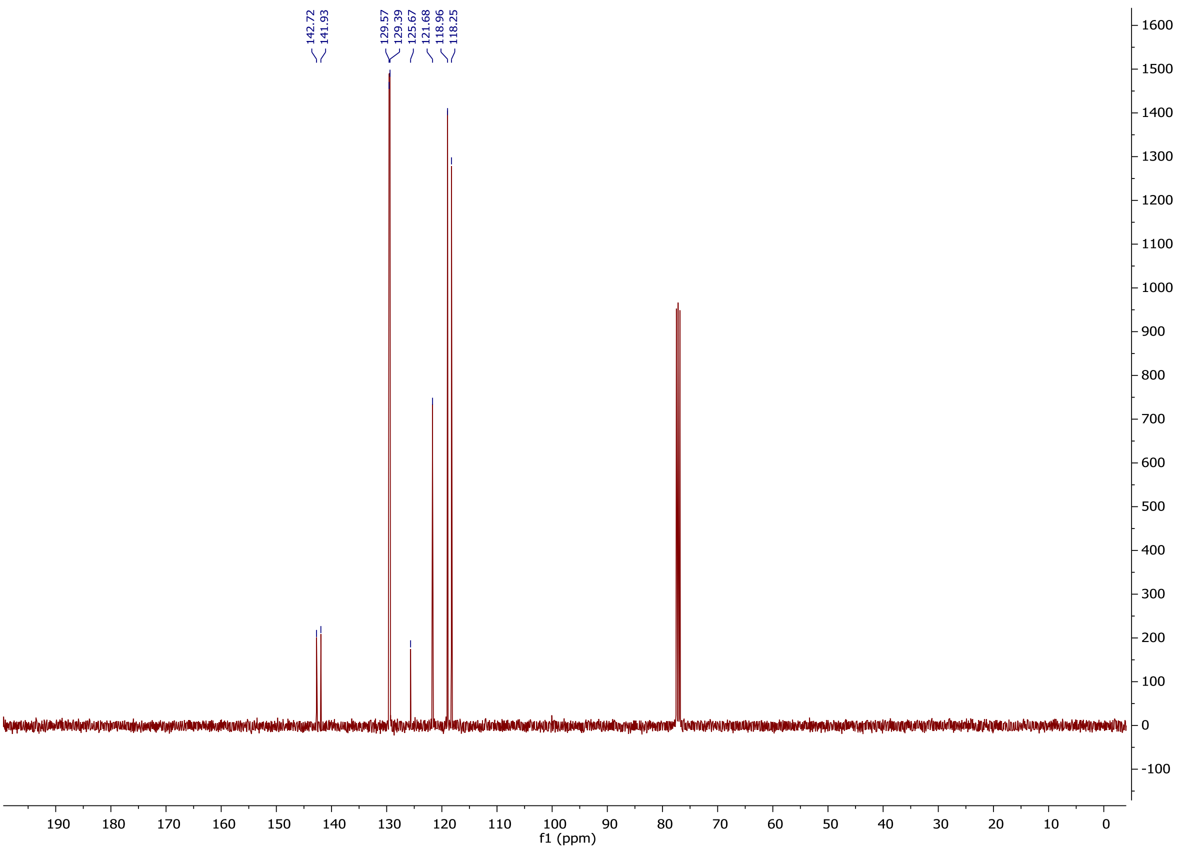

^{1}H NMR (700 MHz, $CDCl_3$) (**25**)

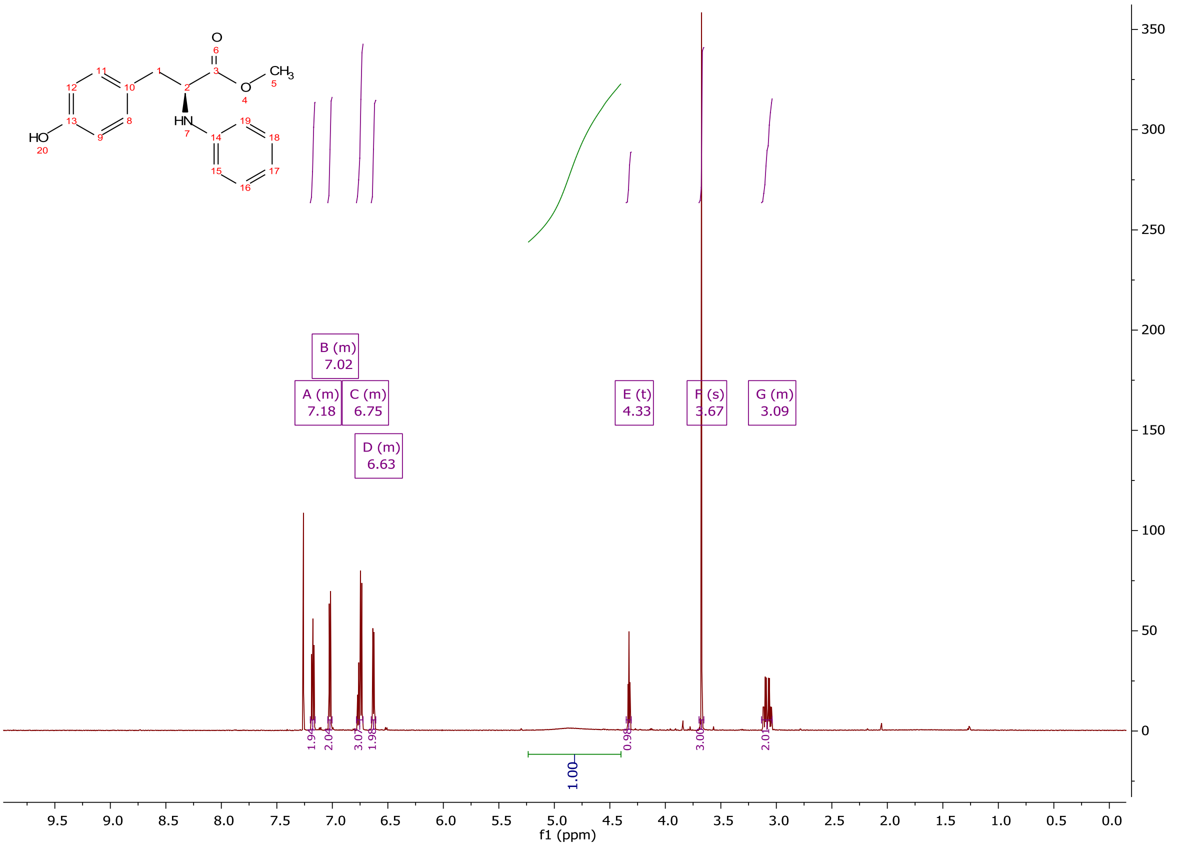

^{13}C NMR (176 MHz, $CDCl_3$) (**25**)

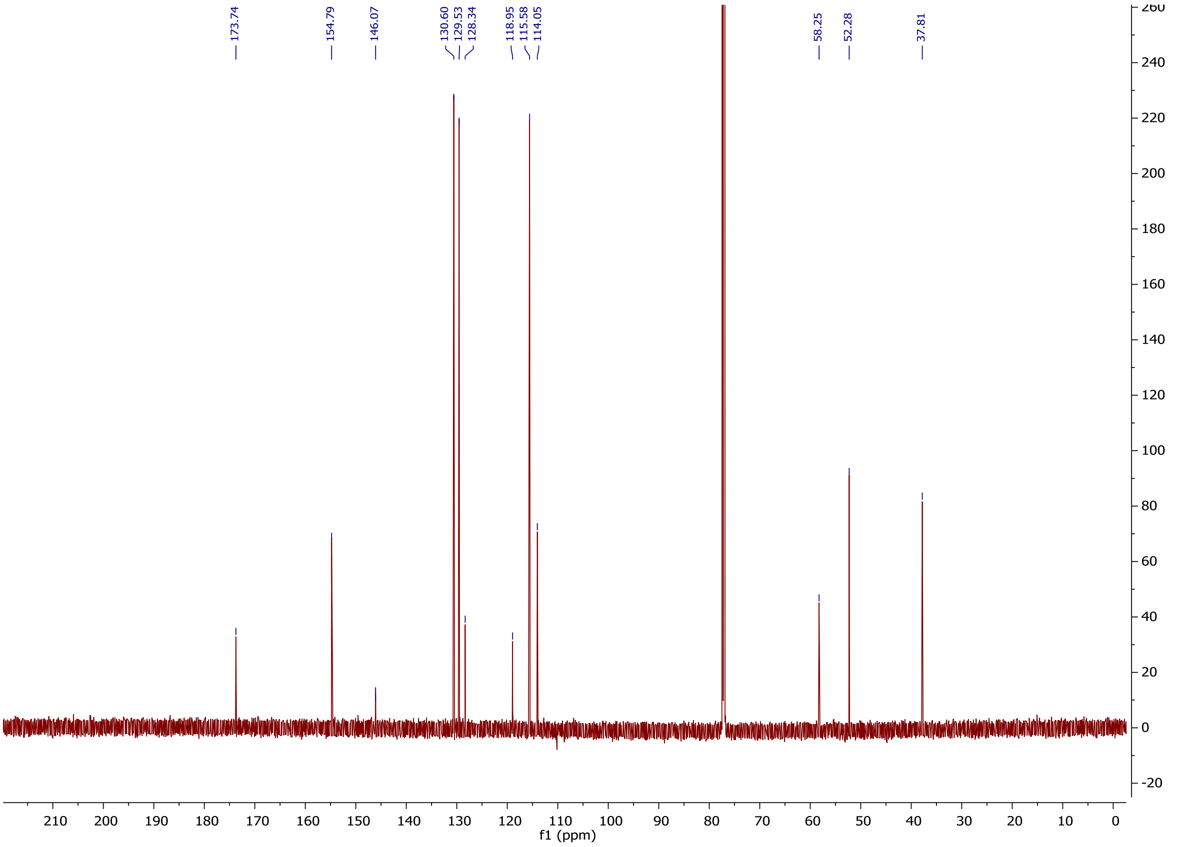

^{1}H *NMR* (700 MHz, $CDCl_3$) (**26**)

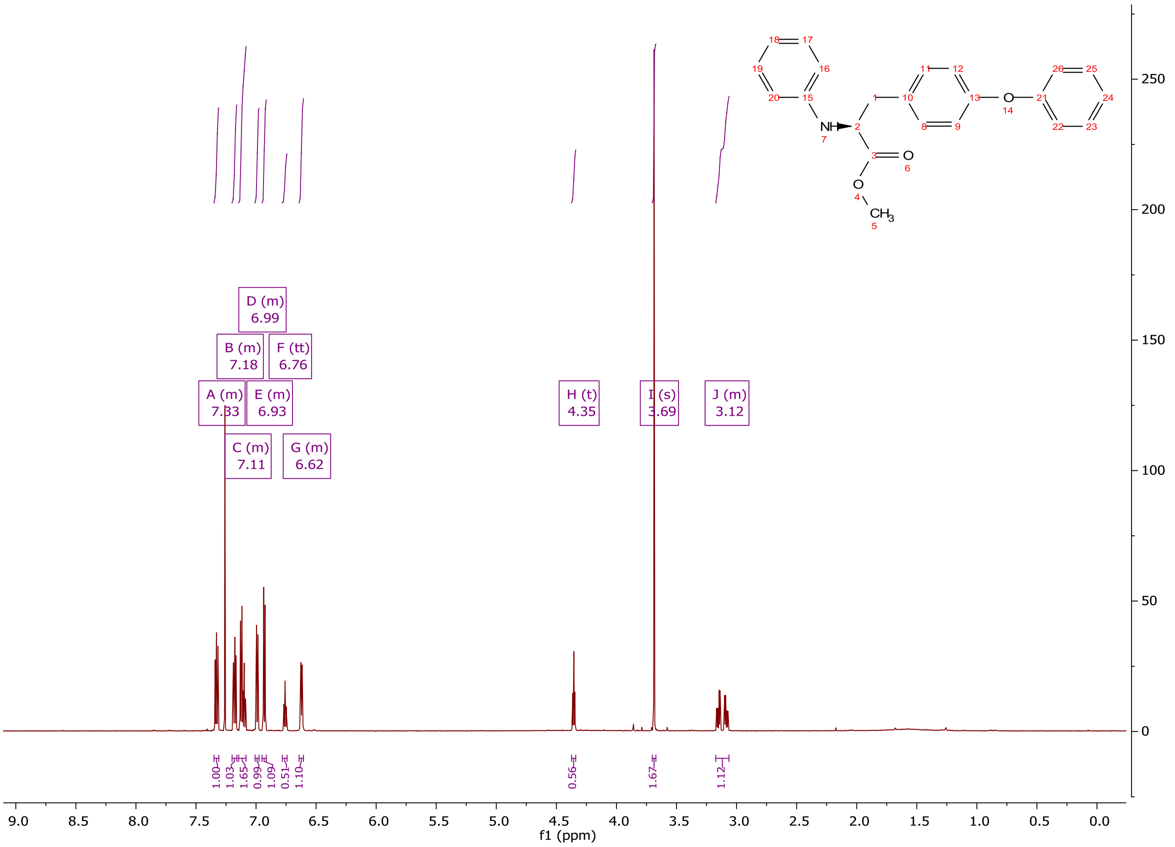

^{13}C *NMR* (176 MHz, $CDCl_3$) (**26**)

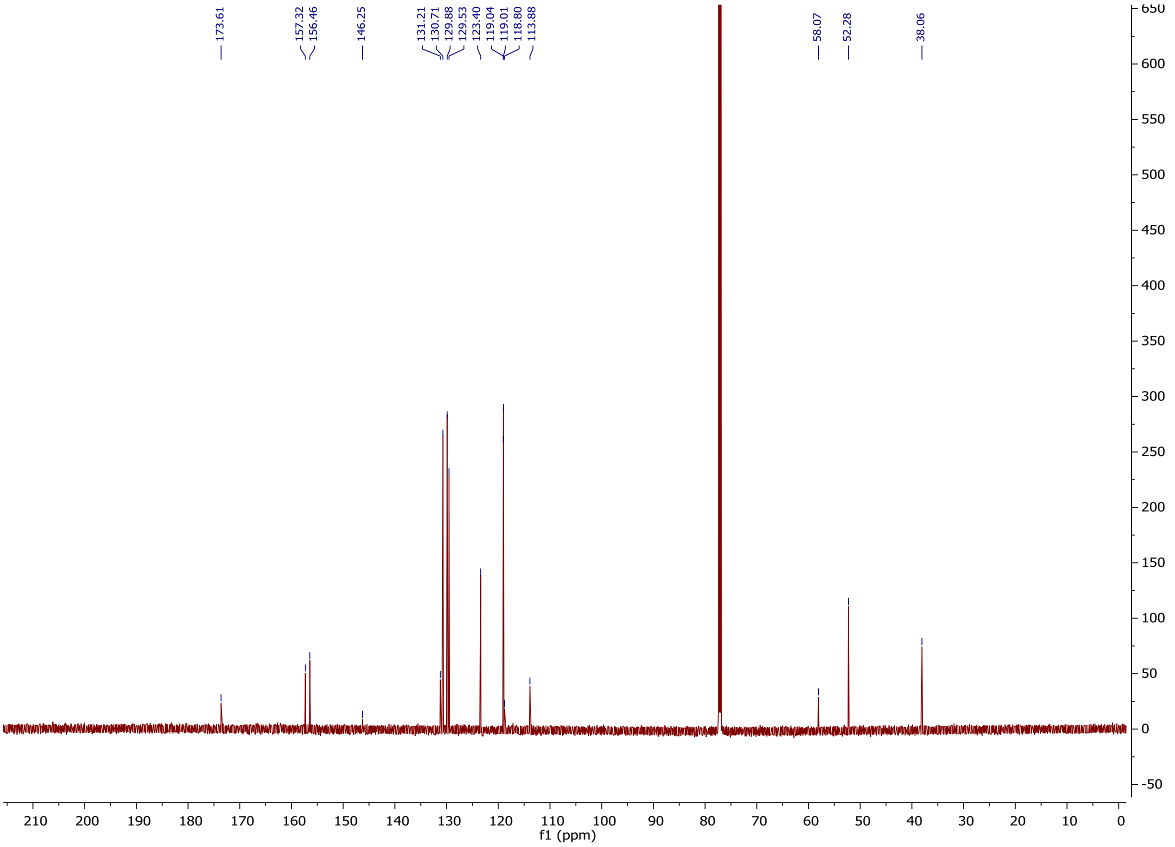

^{1}H *NMR* (400 MHz, $CDCl_3$) (**27**)

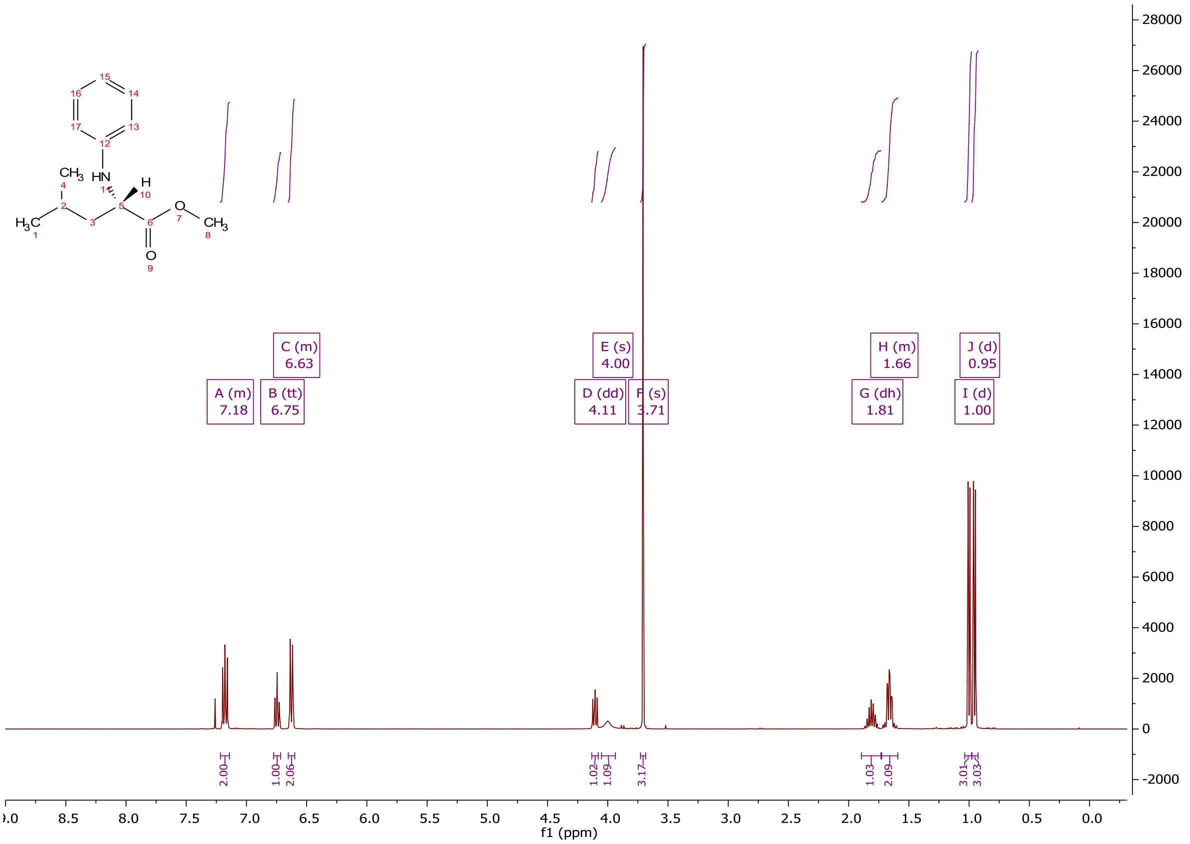

^{13}C *NMR* (101 MHz, $CDCl_3$) (**27**)

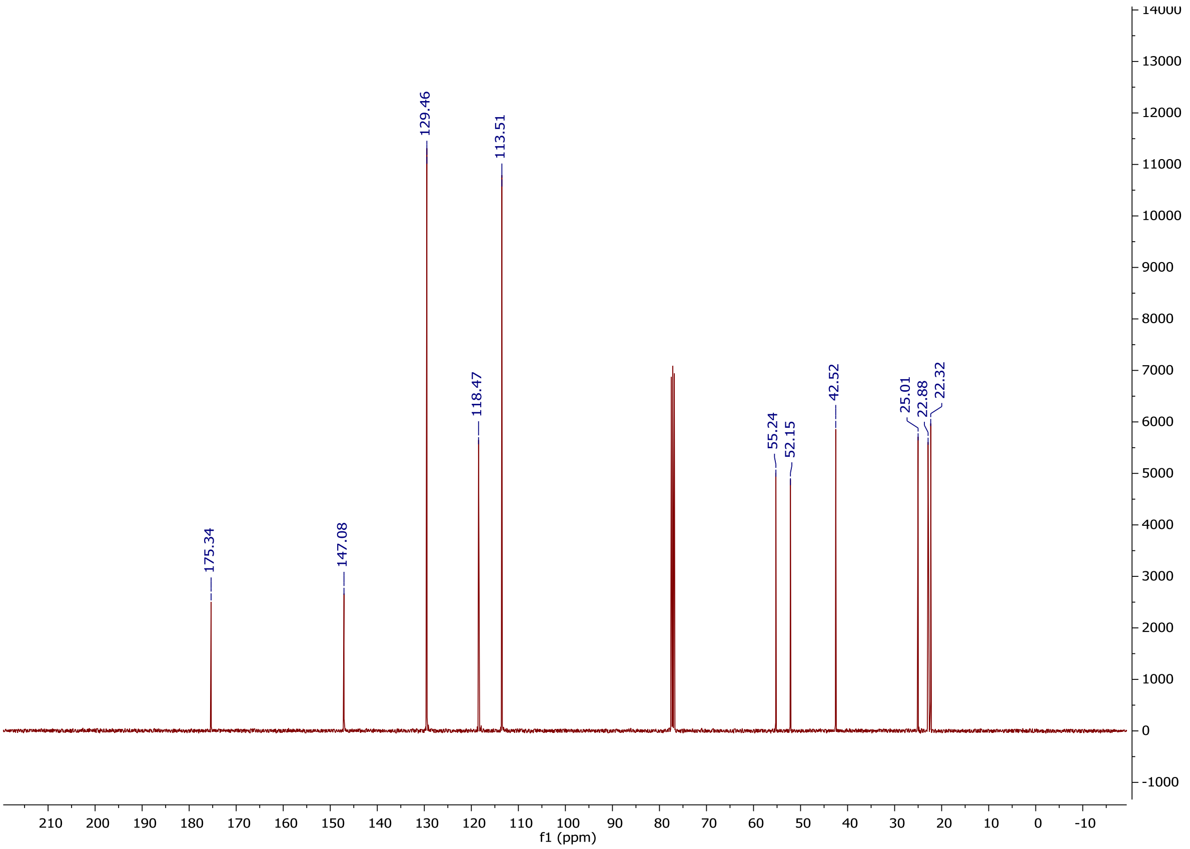

¹H NMR (600 MHz, $CDCl_3$) (**28**)

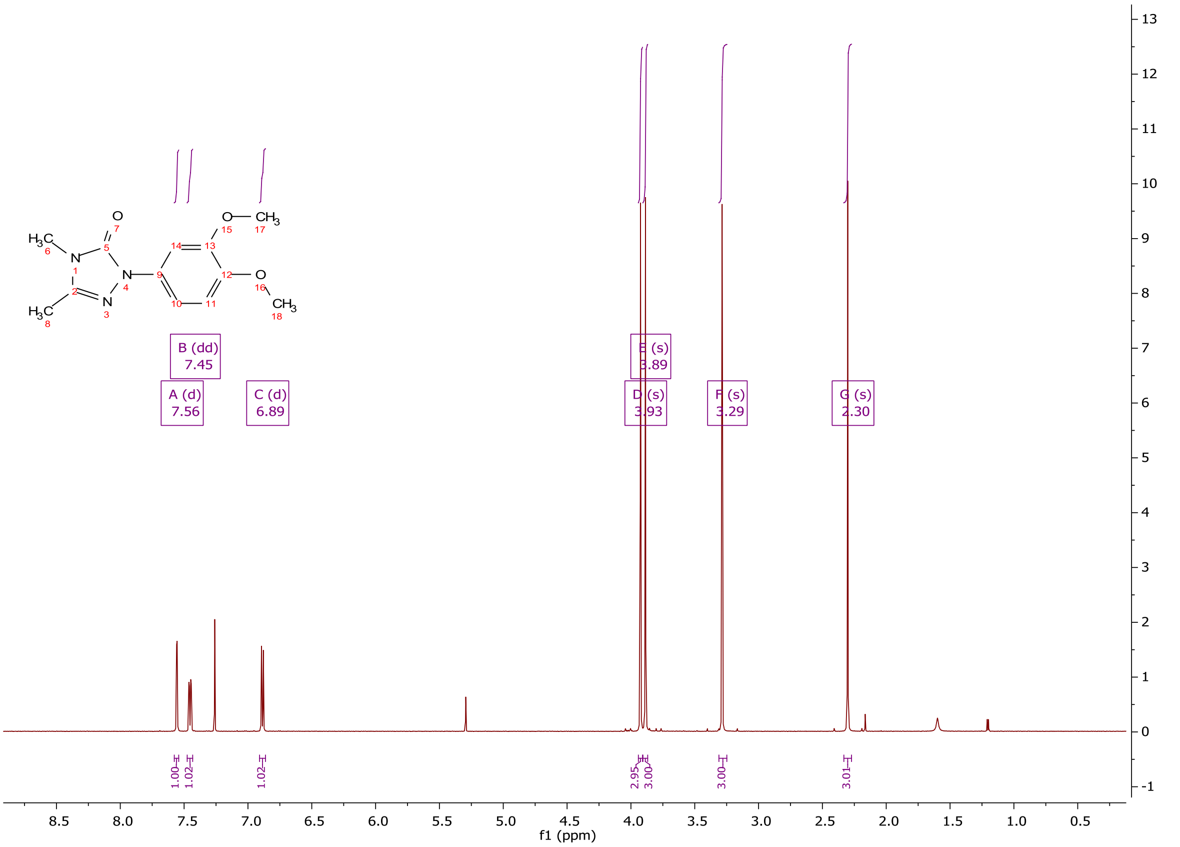

¹³C NMR (151 MHz, $CDCl_3$) (**28**)

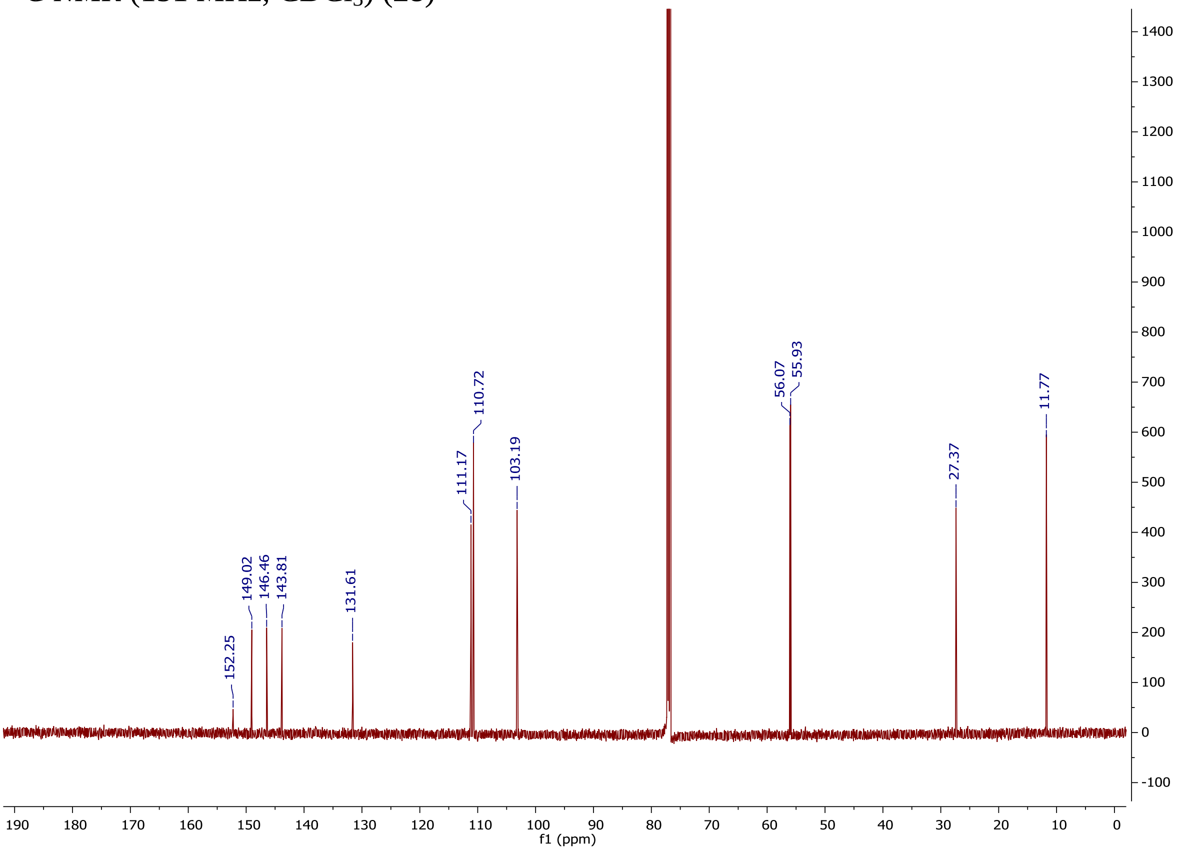

[1]*H NMR* (700 MHz, $CDCl_3$) (**29**)

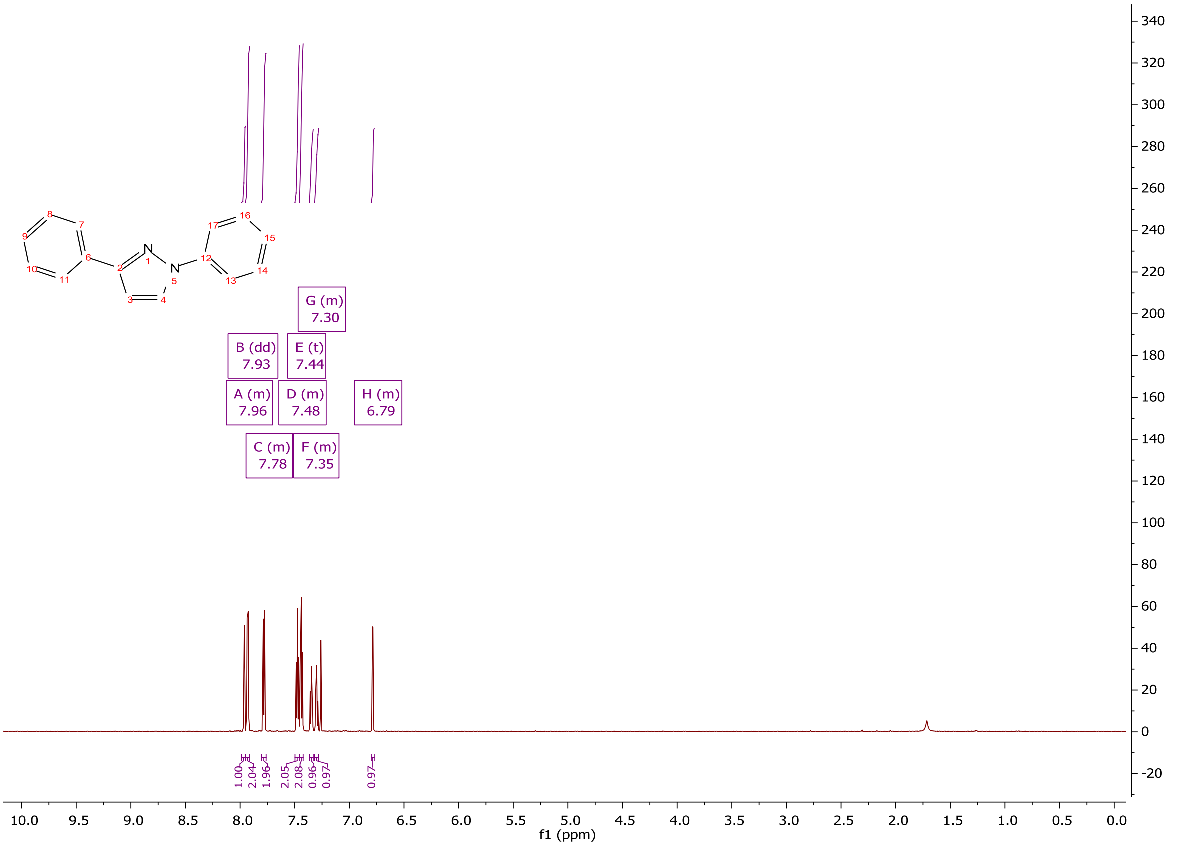

[13]*C NMR* (176 MHz, $CDCl_3$) (**29**)

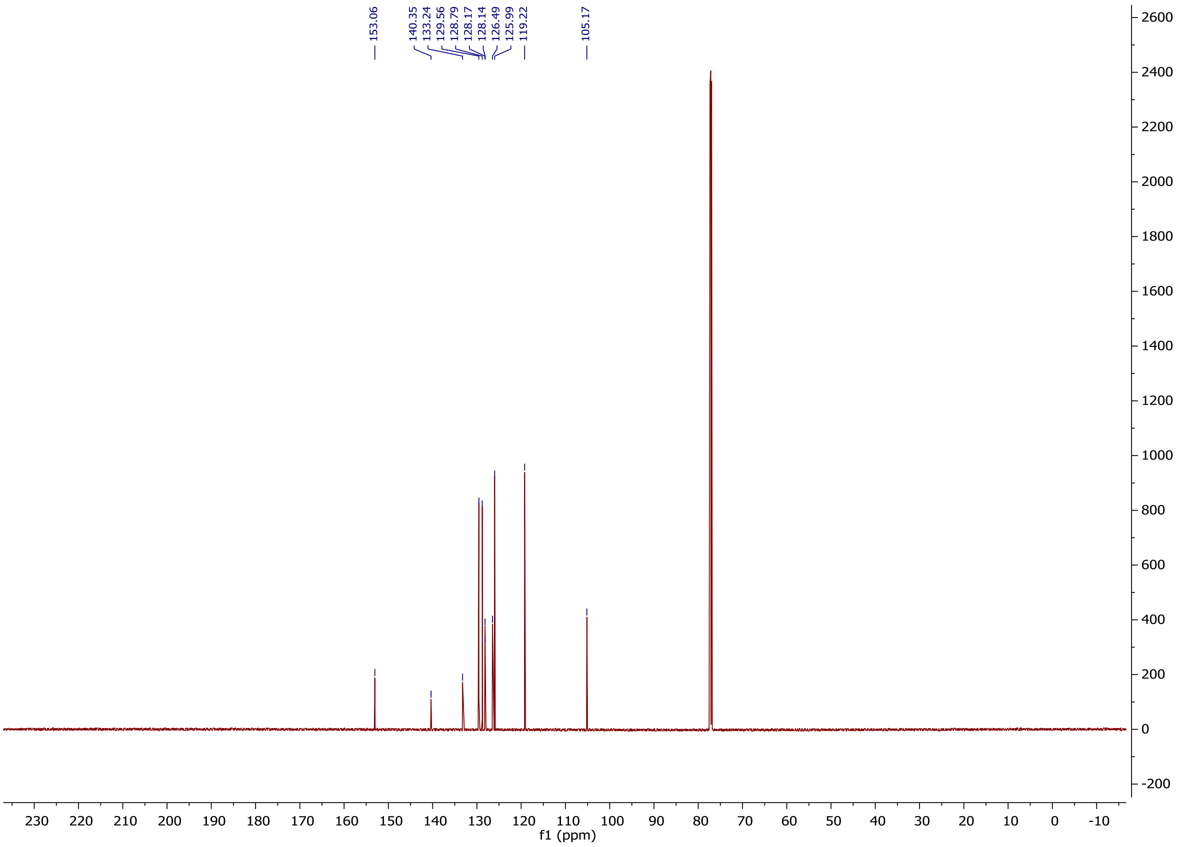

^{1}H NMR (600 MHz, $CDCl_3$) (**30**)

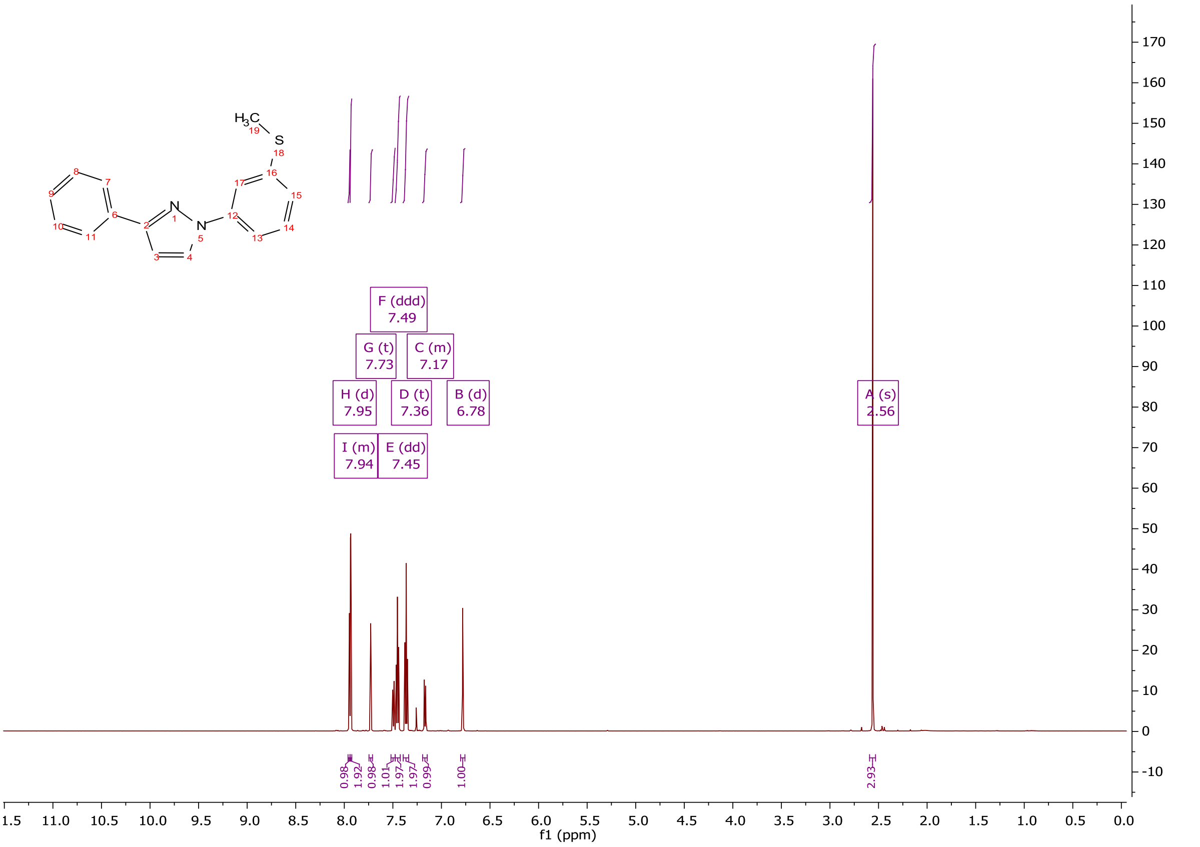

^{13}C NMR (151 MHz, $CDCl_3$) (**30**)

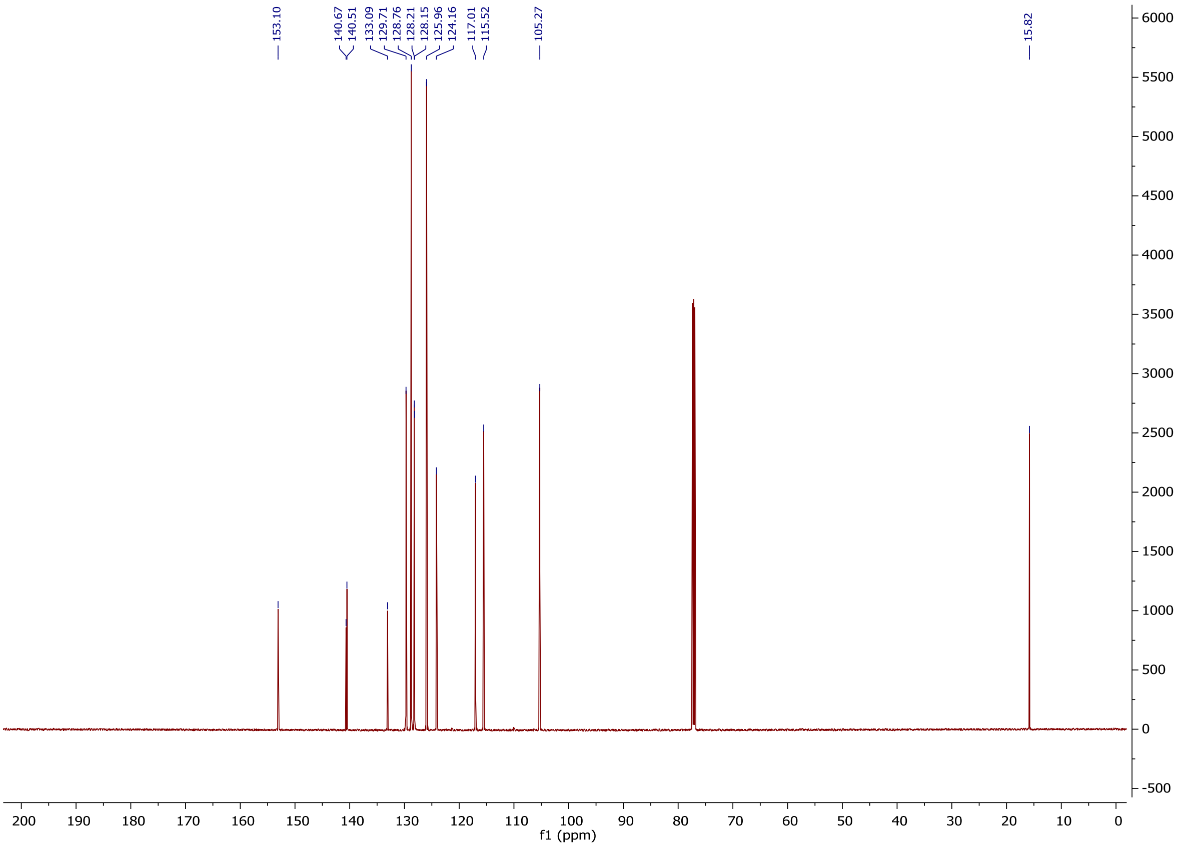

^{1}*H NMR* (600 MHz, $CDCl_3$) (**31**)

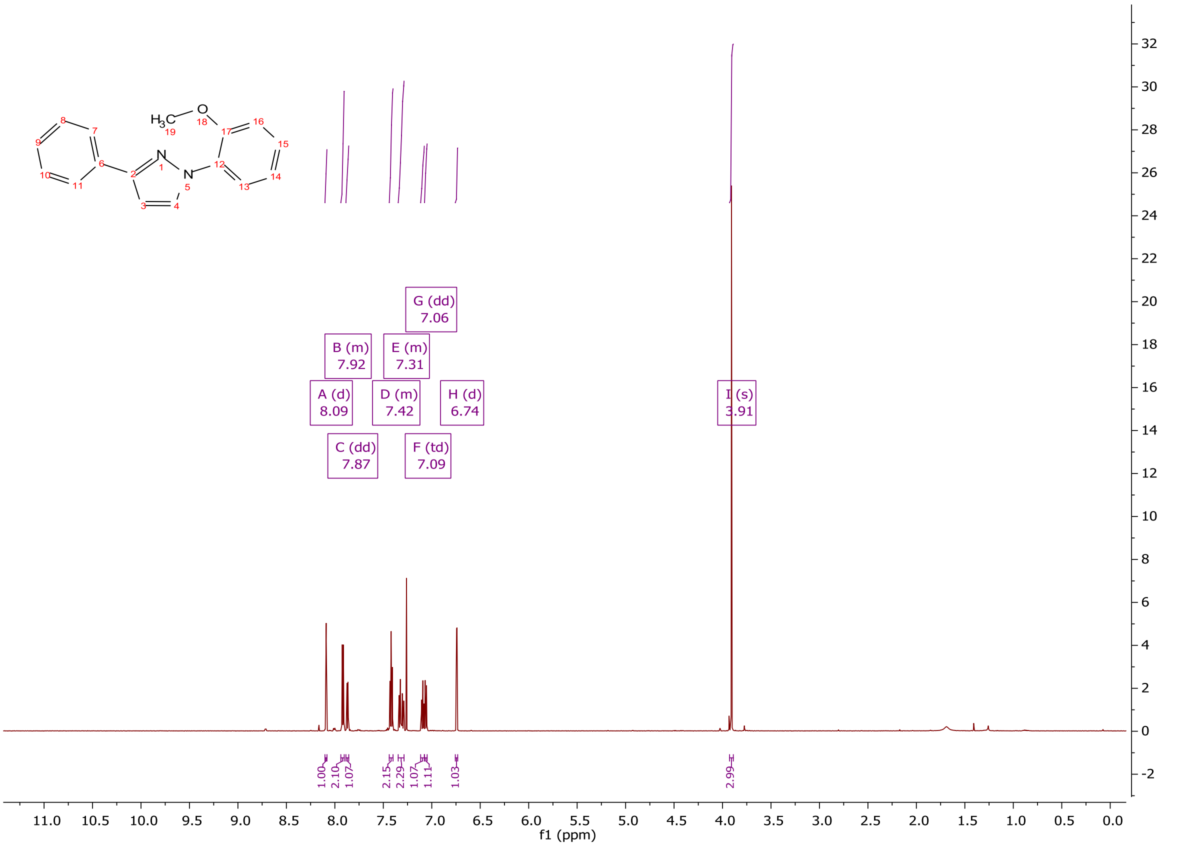

^{13}*C NMR* (151 MHz, $CDCl_3$) (**31**)

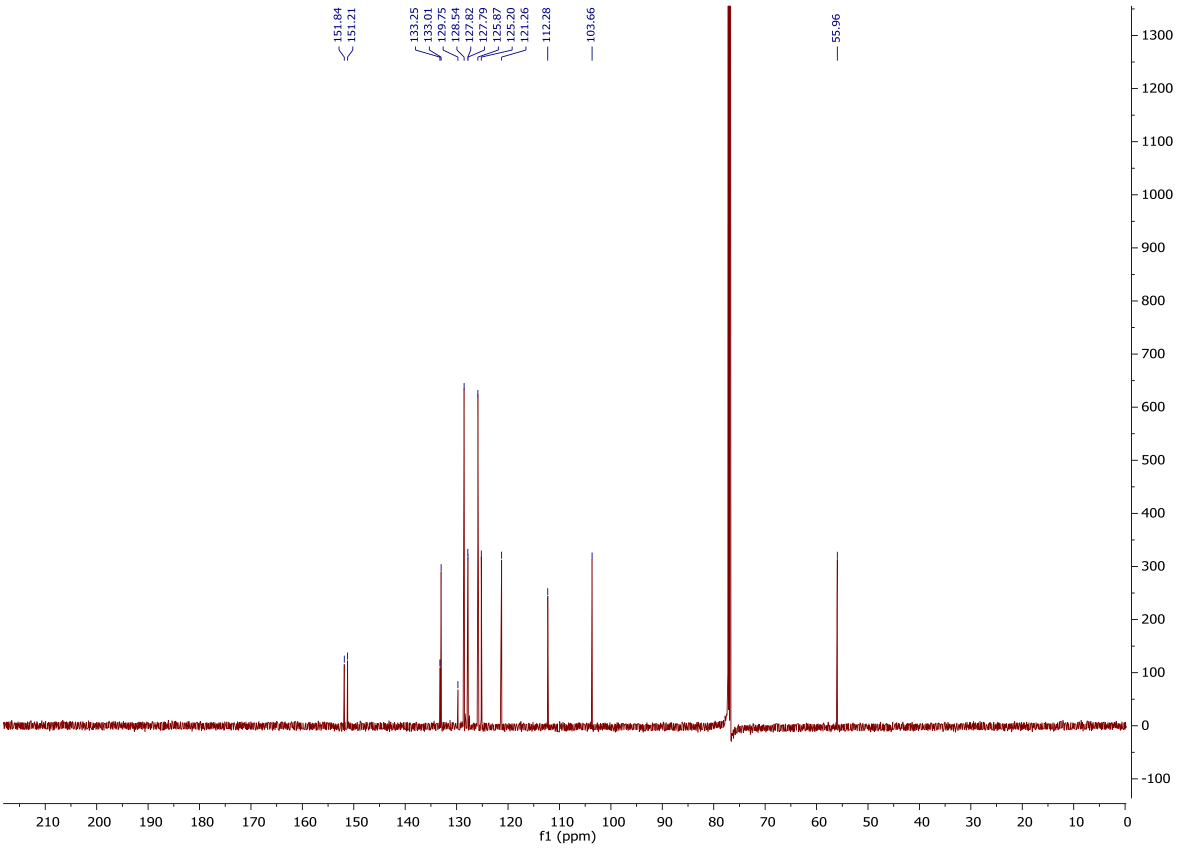

^{1}*H NMR* (400 MHz, $CDCl_3$) (**32**)

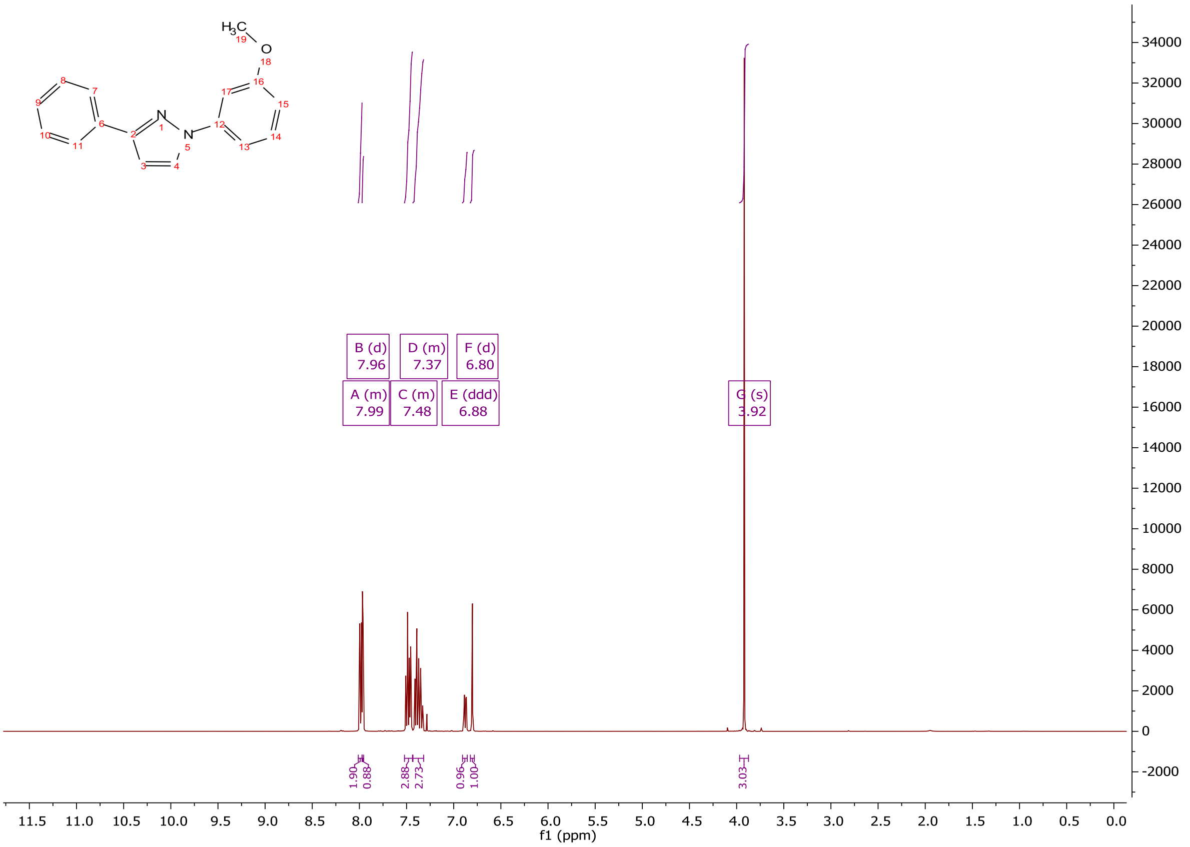

^{13}*C NMR* (101 MHz, $CDCl_3$) (**32**)

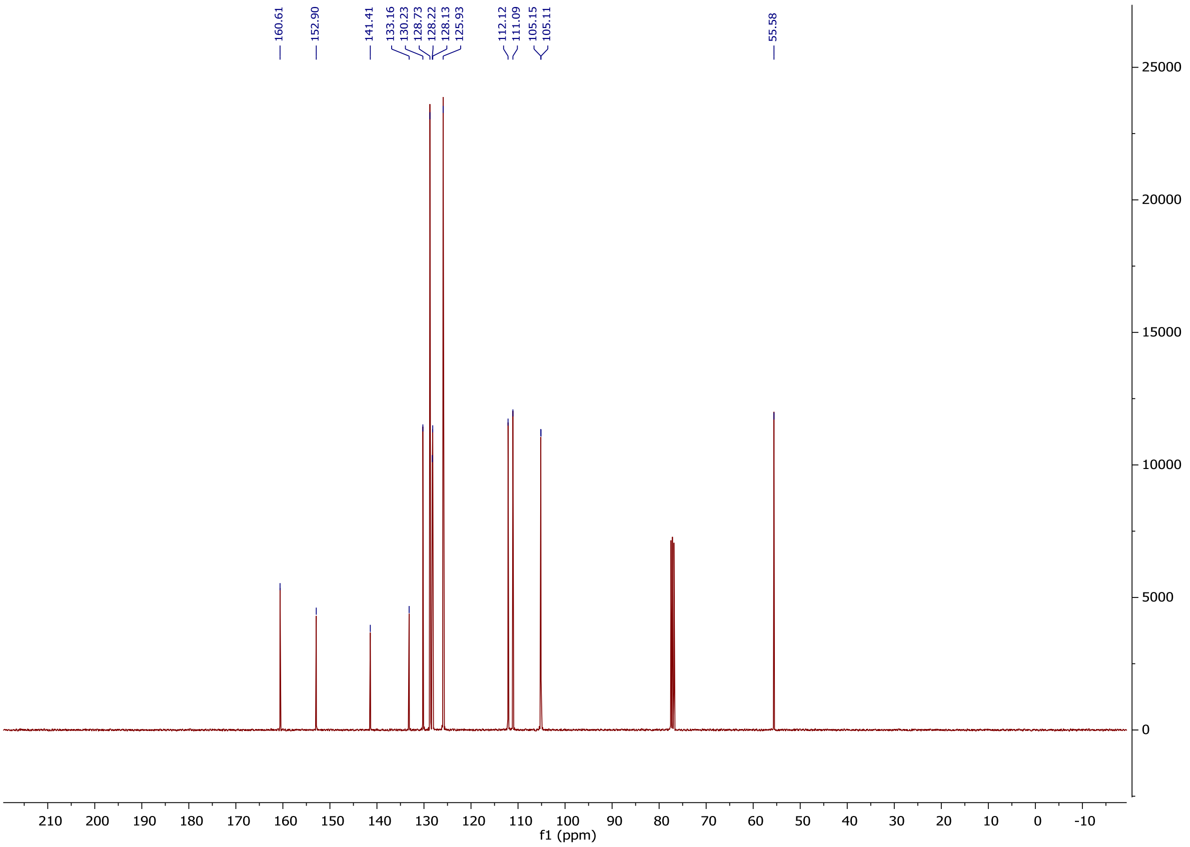

^{1}*H NMR* (700 MHz, $CDCl_3$) (**33**)

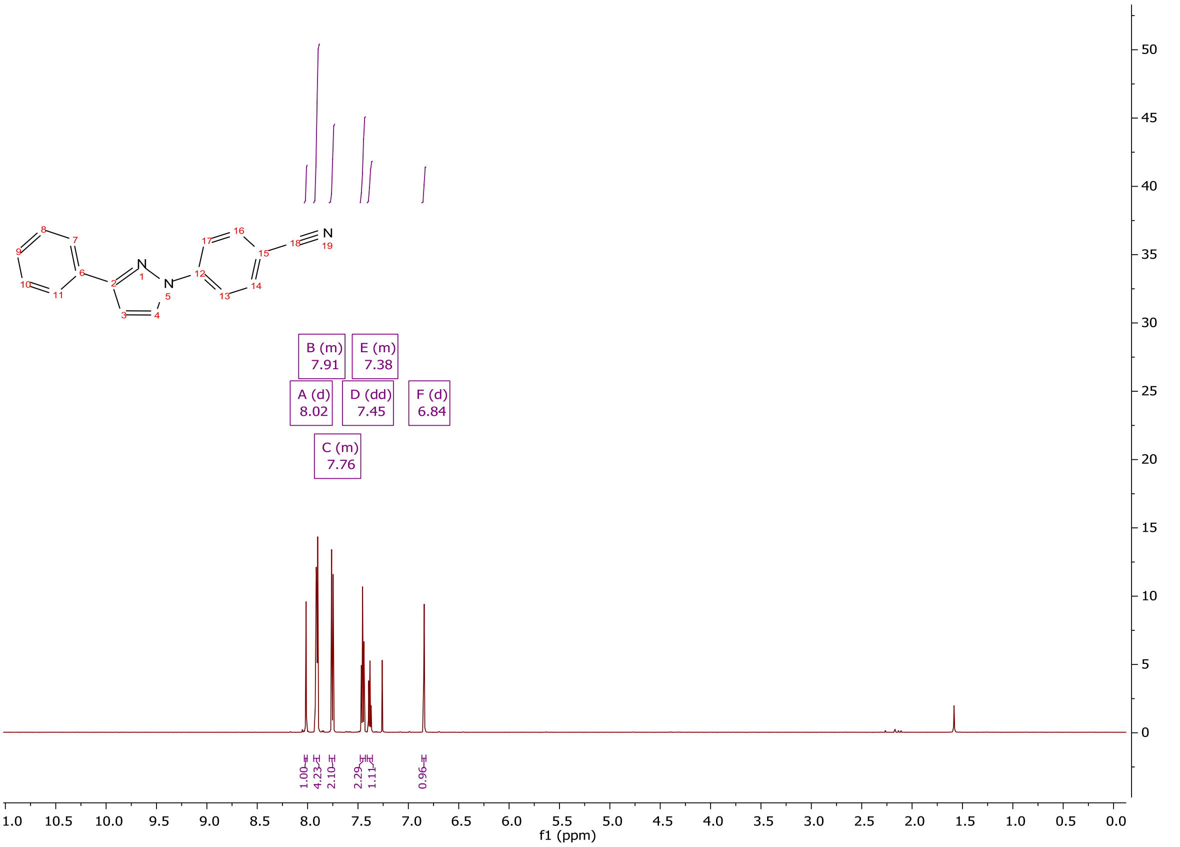

^{13}*C NMR* (176 MHz, $CDCl_3$) (**33**)

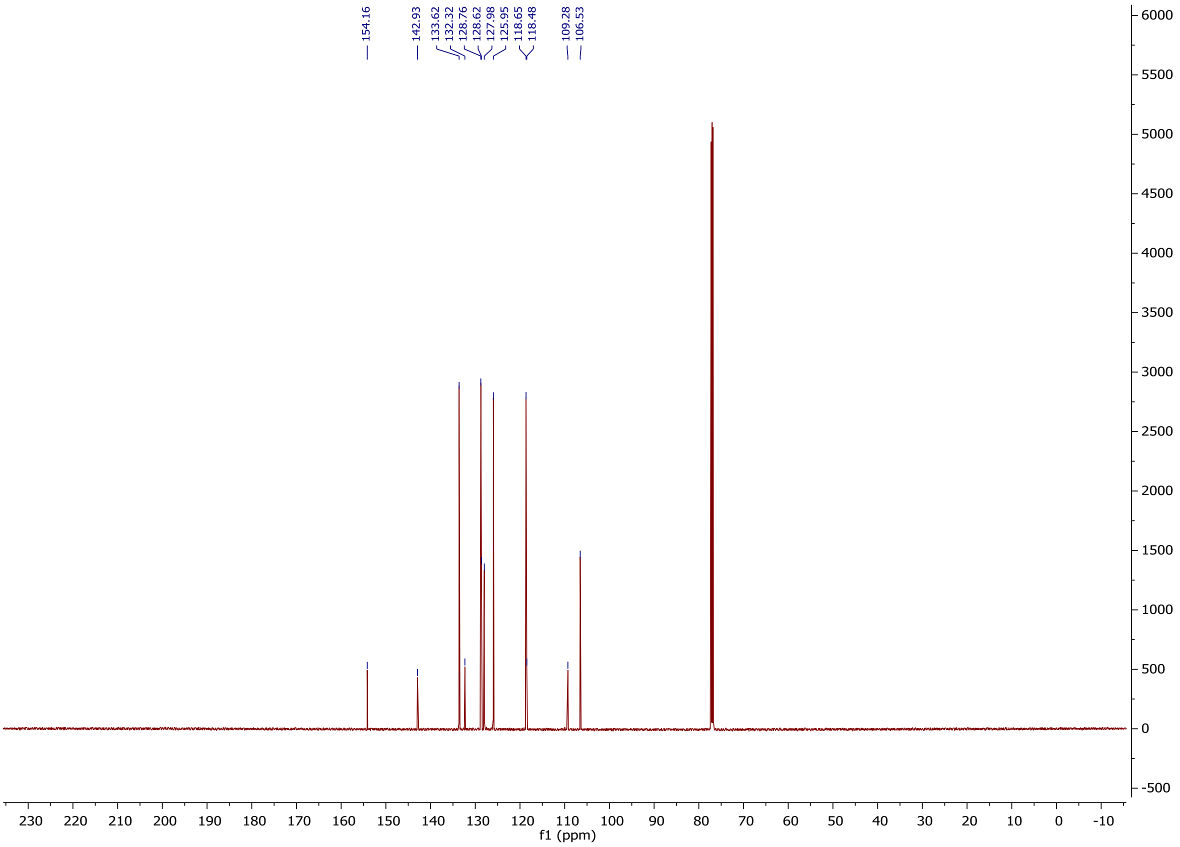

^{1}H NMR (400 MHz, $CDCl_3$) (**34**)

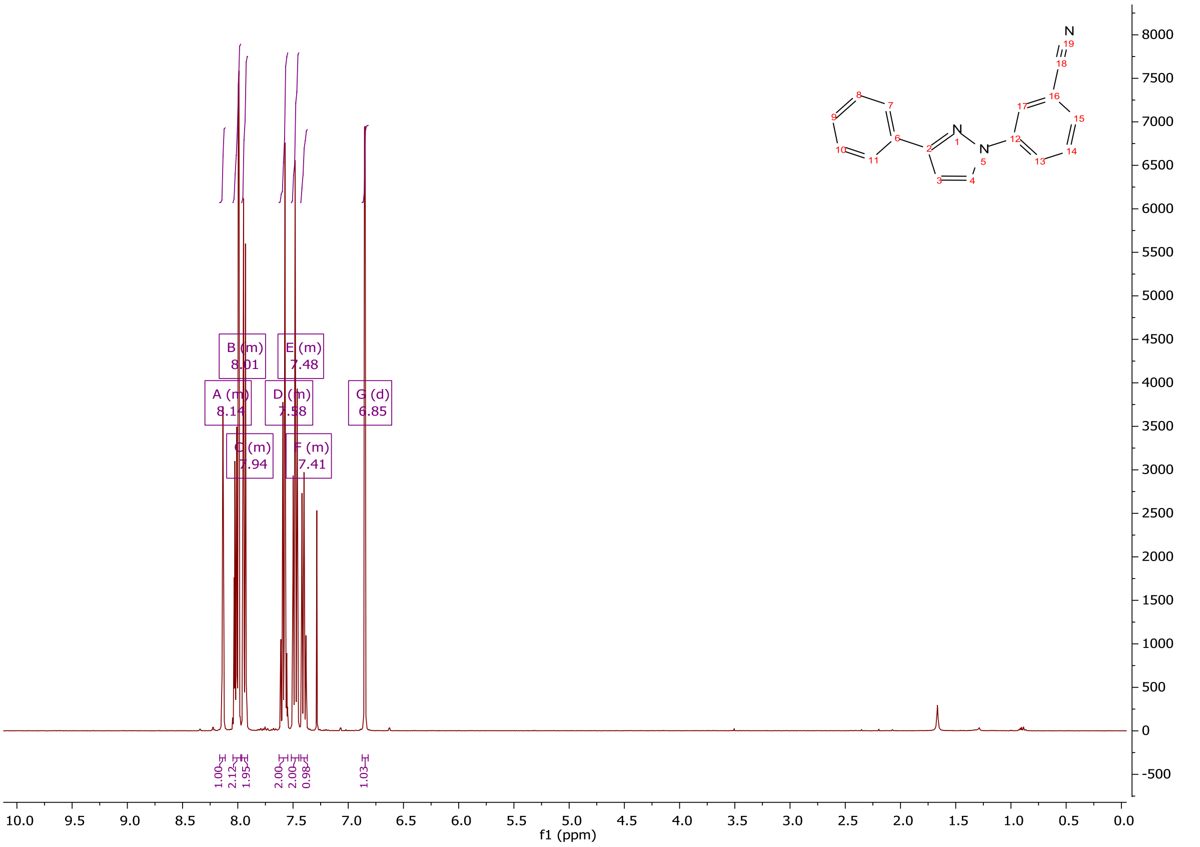

^{13}C NMR (101 MHz, $CDCl_3$) (**34**)

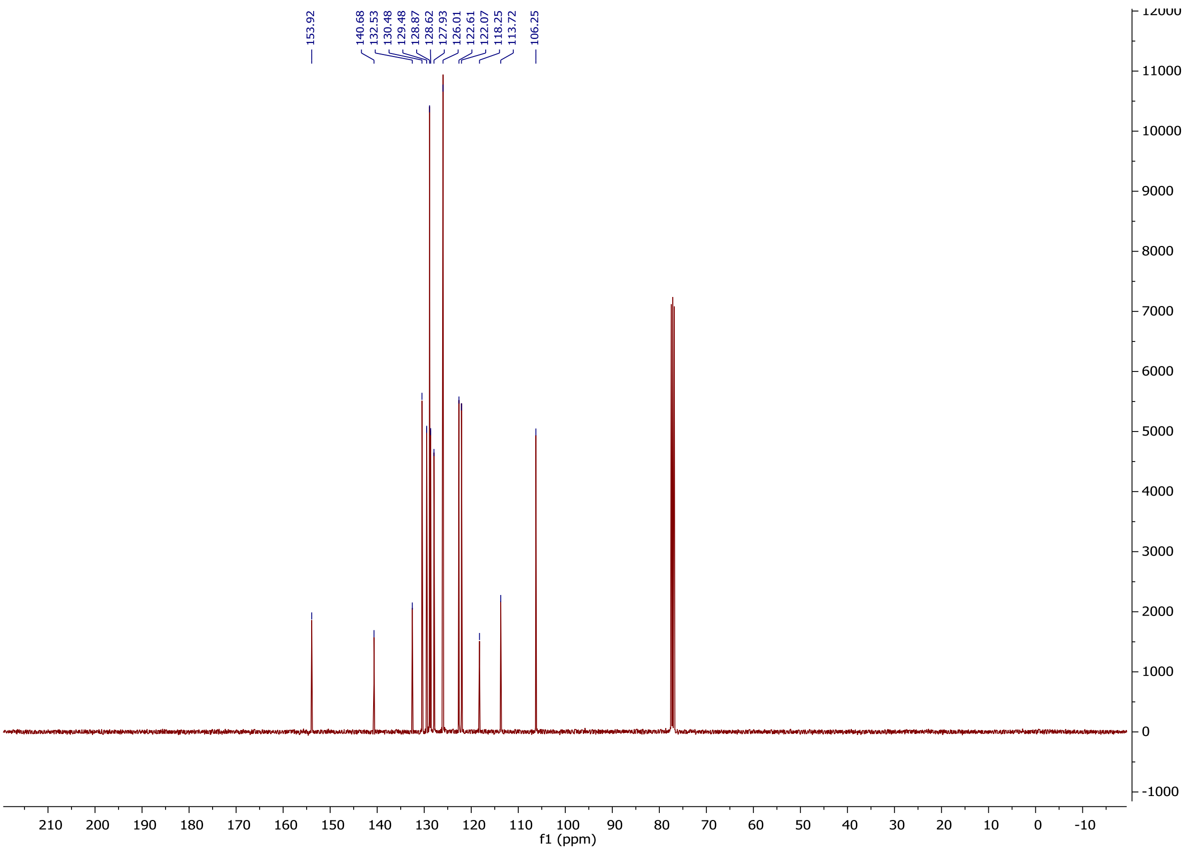

^{1}H NMR (700 MHz, $CDCl_3$) (**35**)

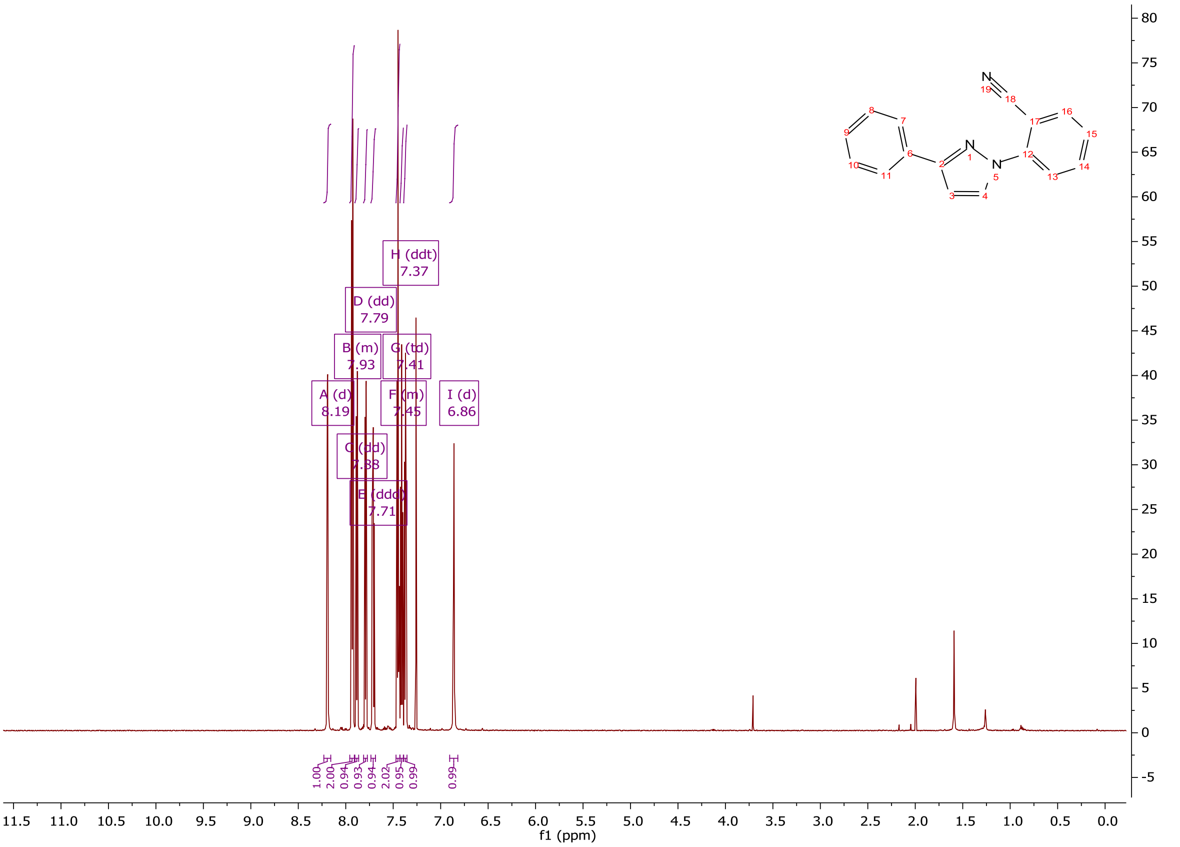

^{13}C NMR (176 MHz, $CDCl_3$) (**35**)

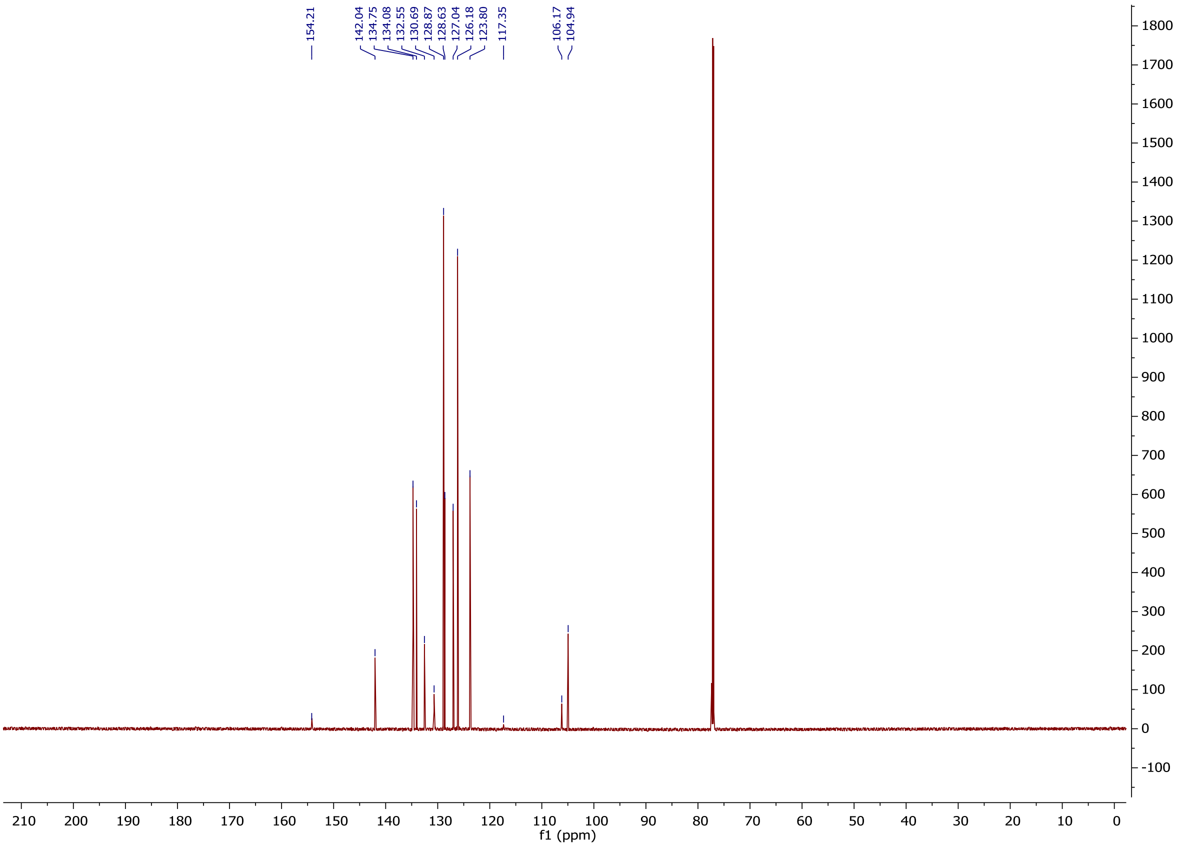

^{1}*H NMR* (400 MHz, $CDCl_3$) (**38**)

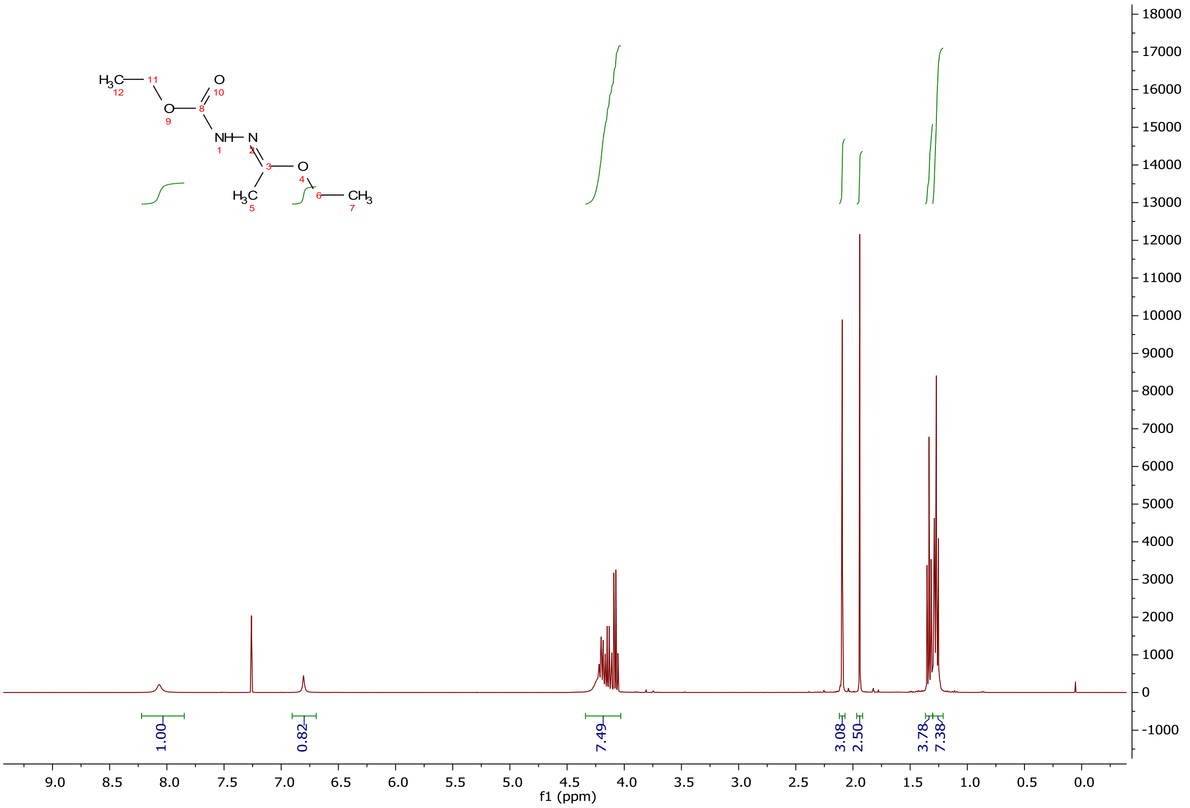

^{13}*C NMR* (101 MHz, $CDCl_3$) (**38**)

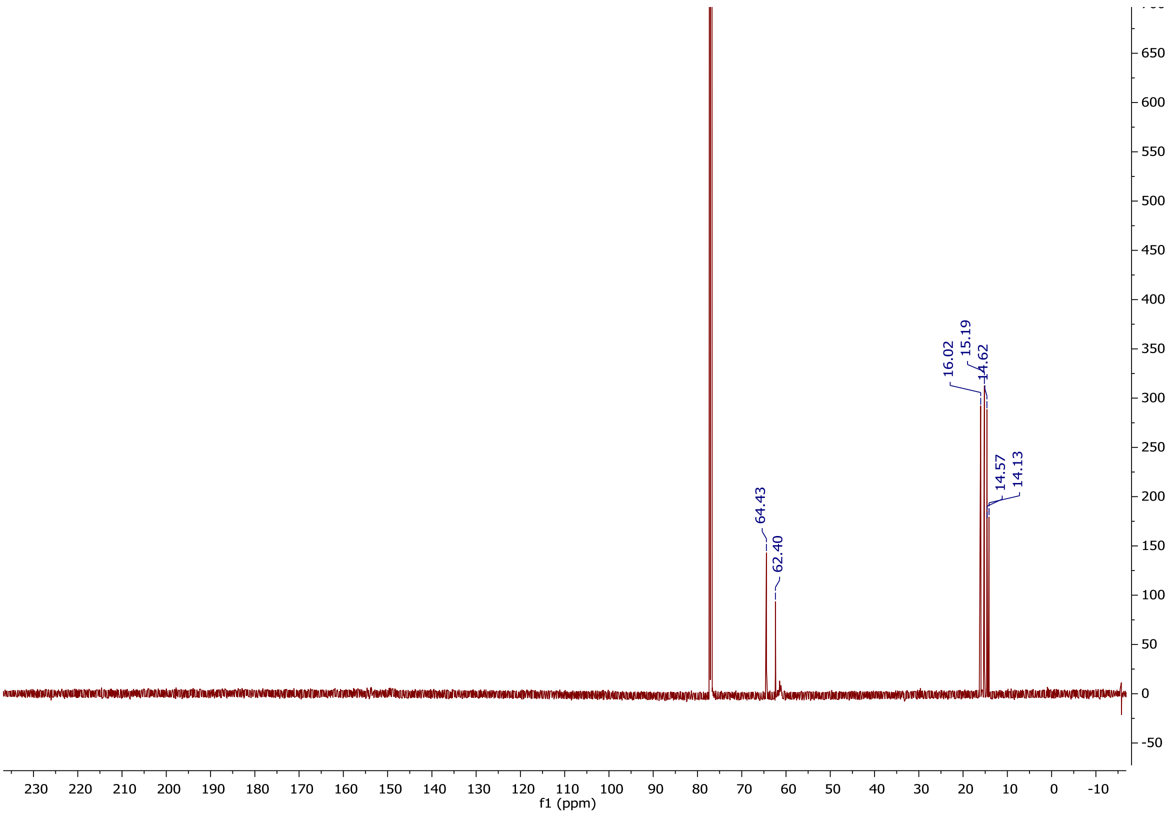

^{1}*H NMR* (600 MHz, $CDCl_3$) (**39**)

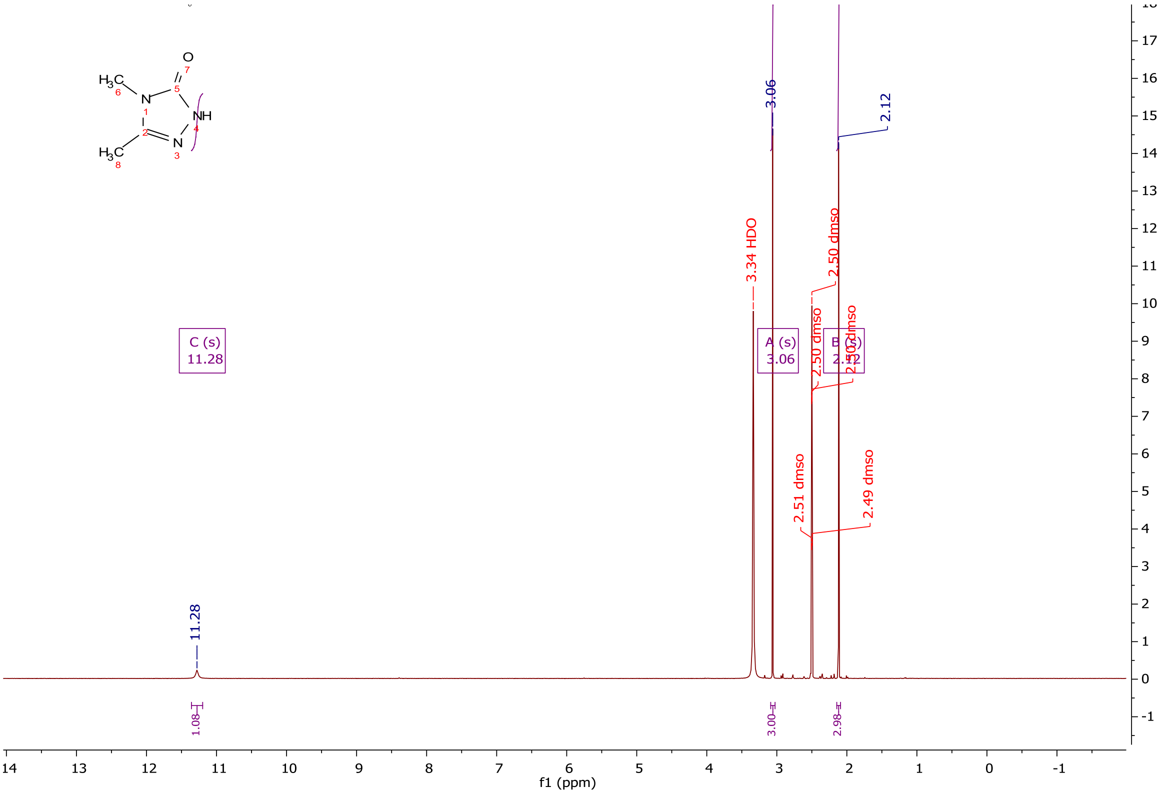

^{13}*C NMR* (151 MHz, $CDCl_3$) (**39**)

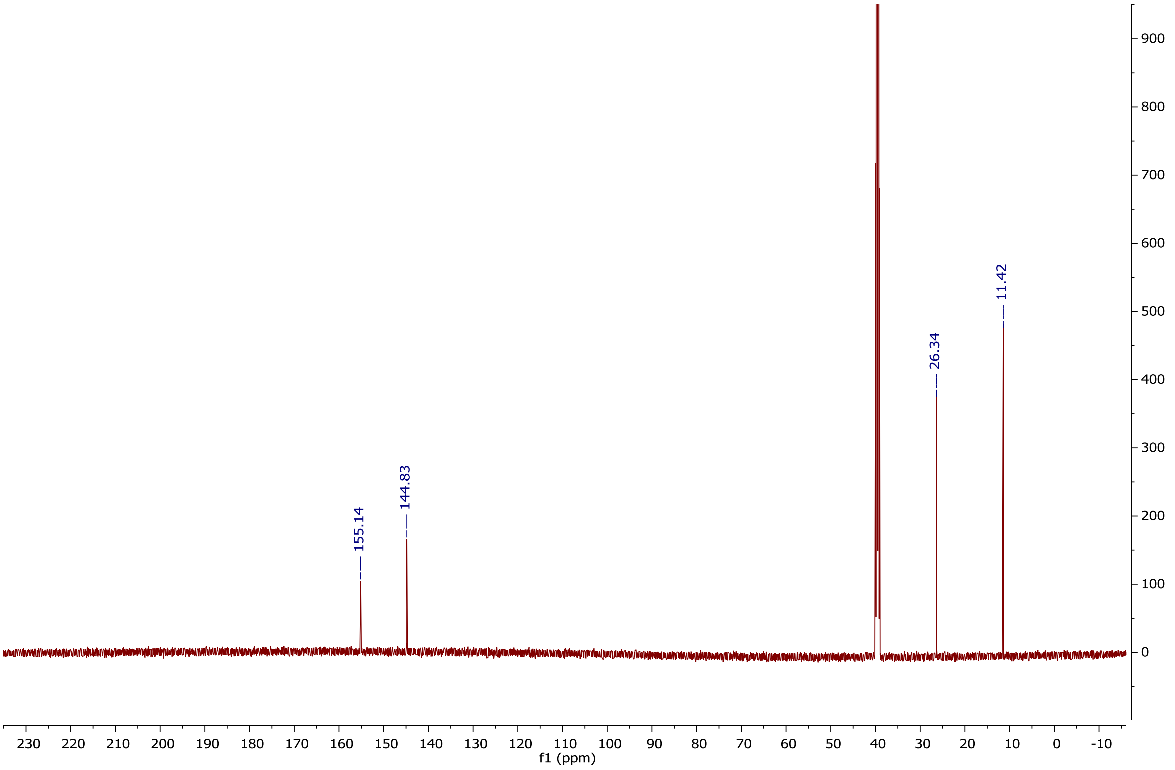

References

1. Gottlieb, H. E.; Kotlyar, V.; Nudelman, A., *J. Org. Chem.* **1997**, *62* (21), 7512-7515.
2. Dolomanov, O. V.; Bourhis, L. J.; Gildea, R. J.; Howard, J. A. K.; Puschmann, H., *J. Appl. Crystallogr.* **2009**, *42* (2), 339-341.
3. Sheldrick, G., *Acta Crystallographica Section A* **2008**, *64* (1), 112-122.
4. Sheldrick, G., *Acta Crystallographica Section A* **2015**, *71* (1), 3-8.
5. Tang, X.; Huang, L.; Yang, J.; Xu, Y.; Wu, W.; Jiang, H., *Chem. Commun.* **2014**, *50* (94), 14793-14796.
6. Alper, H.; Prickett, J. E.; Wollowitz, S., *J. Am. Chem. Soc.* **1977**, *99* (13), 4330-4333.
7. Jadhav, R. R.; Huddar, S. N.; Akamanchi, K. G., *Eur. J. Org. Chem.* **2013**, *2013* (30), 6779-6783.
8. Zhu, X.; Su, L.; Huang, L.; Chen, G.; Wang, J.; Song, H.; Wan, Y., *Eur. J. Org. Chem.* **2009**, *2009* (5), 635-642.
9. Dissanayake, A. A.; Odom, A. L., *Chem. Commun.* **2012**, *48* (3), 440-442.
10. Patra, T.; Agasti, S.; Akanksha; Maiti, D., *Chem. Commun.* **2013**, *49* (1), 69-71.
11. Kahveci, B., *Molecules* **2005**, *10* (2), 376-382.
12. Kahveci, B.; Yılmaz, F.; Menteşe, E.; Ülker, S., *Chemistry of Heterocyclic Compounds* **2015**, *51* (5), 447-456.
13. Ün, R.; Îkizler, A., *Chimica Acta Turcica* **1975**, *3*, 113-132.
